# Computational and functional studies of the PI(4,5)P_2_ binding site of the TRPM3 ion channel reveal interactions with other regulators

**DOI:** 10.1016/j.jbc.2022.102547

**Published:** 2022-09-28

**Authors:** Siyuan Zhao, Vincenzo Carnevale, Matthew Gabrielle, Eleonora Gianti, Tibor Rohacs

**Affiliations:** 1 Department of Pharmacology, Physiology and Neuroscience, Rutgers, New Jersey Medical School, Newark, New Jersey, USA; 2 Institute for Computational Molecular Science, Temple University, Philadelphia, Pennsylvania, USA; 3 Department of Biology, Temple University, Philadelphia, Pennsylvania, USA; 4 Institute for Genomics and Evolutionary Medicine, Temple University, Philadelphia, Pennsylvania, USA; 5 Department of Chemistry, Temple University, Philadelphia, Pennsylvania, USA

**Keywords:** TRPM3, TRP channel, ion channel, phosphoinositide, computational modeling, fcTRPM8, flycatcher apo TRPM8, hM2, human M2 muscarinic, hTRPM3, human TRPM3, MM/GBSA, molecular mechanics/generalized Born surface area, mTRPM3α2, mouse TRPM3α2, PDB, Protein Data Bank, PI(4,5)P_2_, phosphatidylinositol 4,5-bisphosphate, PregS, pregnenolone sulfate, TEVC, two-electrode voltage clamp, TRPM3, transient receptor potential melastatin 3, TRPV1, transient receptor potential vanilloid 1, VMD, Visual Molecular Dynamics

## Abstract

Transient receptor potential melastatin 3 (TRPM3) is a heat-activated ion channel expressed in peripheral sensory neurons and the central nervous system. TRPM3 activity depends on the membrane phospholipid phosphatidylinositol 4,5-bisphosphate (PI(4,5)P_2_), but the molecular mechanism of activation by PI(4,5)P_2_ is not known. As no experimental structure of TRPM3 is available, we built a homology model of the channel in complex with PI(4,5)P_2_*via* molecular modeling. We identified putative contact residues for PI(4,5)P_2_ in the pre-S1 segment, the S4–S5 linker, and the proximal C-terminal TRP domain. Mutating these residues increased sensitivity to inhibition of TRPM3 by decreasing PI(4,5)P_2_ levels. Changes in ligand-binding affinities *via* molecular mechanics/generalized Born surface area (MM/GBSA) showed reduced PI(4,5)P_2_ affinity for the mutants. Mutating PI(4,5)P_2_-interacting residues also reduced sensitivity for activation by the endogenous ligand pregnenolone sulfate, pointing to an allosteric interaction between PI(4,5)P_2_ and pregnenolone sulfate. Similarly, mutating residues in the PI(4,5)P_2_ binding site in TRPM8 resulted in increased sensitivity to PI(4,5)P_2_ depletion and reduced sensitivity to menthol. Mutations of most PI(4,5)P_2_-interacting residues in TRPM3 also increased sensitivity to inhibition by Gβγ, indicating allosteric interaction between Gβγ and PI(4,5)P_2_ regulation. Disease-associated gain-of-function TRPM3 mutations on the other hand resulted in no change of PI(4,5)P_2_ sensitivity, indicating that mutations did not increase channel activity *via* increasing PI(4,5)P_2_ interactions. Our data provide insight into the mechanism of regulation of TRPM3 by PI(4,5)P_2_, its relationship to endogenous activators and inhibitors, as well as identify similarities and differences between PI(4,5)P_2_ regulation of TRPM3 and TRPM8.

Transient receptor potential melastatin 3 (TRPM3) is a heat-activated, outwardly rectifying, and Ca^2+^-permeable nonselective cation channel expressed in a variety of tissues, including peripheral sensory neurons of the dorsal root ganglia, and the central nervous system (1). Its chemical activators include the endogenous neurosteroid pregnenolone sulfate (PregS) (2) and the synthetic compound CIM0216 (3). TRPM3 activity can be inhibited by a number of compounds, including natural flavonones, such as isosakuranetin (4), the nonsteroid anti-inflammatory drug diclofenac, and the antiepileptic medication primidone (5). TRPM3 is a very well-established peripheral noxious heat sensor. Genetic deletion of this channel in mice results in impaired noxious heat sensation (6, 7, 8) and impaired inflammatory thermal hyperalgesia (6, 8). TRPM3 inhibitors also reduce thermal hyperalgesia and basal heat sensitivity (4, 5, 8).

Activation of Gi-coupled receptors inhibits TRPM3 activity. This effect was demonstrated both by native receptors in dorsal root ganglia neurons, including μ-opioid and GABAB receptors (9, 10, 11), as well as by heterologously expressing Gi-coupled receptors such as M2 muscarinic receptor and μ-opioid receptors (9, 10). Inhibition by Gi-coupled receptors is mediated by direct binding of Gβγ to the channel protein (9, 10, 11) through a short α-helical peptide encoded by an alternatively spliced exon in TRPM3, the costructure of which with Gβγ has been recently determined by X-ray crystallography (12). (TRPM3 has a large number of splice variants, and some of the alternatively spliced exons are in the N terminus (1), which makes residue numbering confusing (13, 14, 15)). The Gβγ binding peptide is present in TRPM1, the closest relative of TRPM3, which is also inhibited by Gβγ (16), but it is missing from the rest of the TRPM family. Activation of recombinant (9) or native (17) Gq-coupled receptors may also inhibit TRPM3, which is also mediated mainly by Gβγ binding (9).

It was recently shown that mutations in TRPM3 are associated with developmental and epileptic encephalopathies manifesting as intellectual disability and seizures in children (13). The originally described two disease-associated mutations both showed a gain-of-function phenotype with increased basal activity and increased heat and agonist sensitivity (14, 15). This points to the importance of TRPM3 in the brain, but knowledge on the functional role of TRPM3 in the central nervous system is quite limited (18).

Phosphoinositides, especially PI(4,5)P_2_, are common ion channel regulators (19, 20). Most TRP channels, including TRPM3 (21, 22), are positively regulated by phosphoinositides (23), but in some cases such as transient receptor potential vanilloid 1 (TRPV1) (24, 25), or transient receptor potential canonical channels (TRPC) (26, 27), this regulation is complex, and sometimes controversial, with both negative and positive effects having been proposed. With the exception of TRPM1, which is very difficult to study in expression systems, all members of the TRPM subfamily have been shown to be positively regulated by PI(4,5)P_2,_ and no negative regulation has been proposed for any TRPM subfamily member (23). While cryo-EM structures are available for five of eight members, the only TRPM channel for which the PI(4,5)P_2_ binding site is revealed by structural studies is TRPM8 (28).

Currently, it is not known which residues in the TRPM3 protein PI(4,5)P_2_ binds to, and there is no experimentally determined structure available for TRPM3. To fill this key gap in knowledge, we generated a homology model of TRPM3, based on the experimental structure of TRPM4 in the ligand-free (apo) state (29). We then docked PI(4,5)P_2_ to our model of TRPM3 and identified putative PI(4,5)P_2_-interacting residues in the pre-S1 segment, the S4–S5 linker, and the proximal C-terminal TRP domain. We validated our results by docking PI(4,5)P_2_ to an apo structure of TRPM8 (30), which showed remarkable similarity to the TRPM8–PI(4,5)P_2_ structures (28), experimentally determined recently. *In silico* mutations of the PI(4,5)P_2_ contact residues in TRPM3, followed by ligand-binding affinity changes *via* molecular MM/GBSA, showed reduced PI(4,5)P_2_ binding affinity to TRPM3. We experimentally validated the importance of these residues by demonstrating that their mutations increased sensitivity to inhibition by PI(4,5)P_2_ depletion in electrophysiology experiments. We also showed that mutating most of these residues increased sensitivity to Gβγ inhibition, and decreased sensitivity to agonist activation, indicating allosteric interaction between PI(4,5)P_2_ and endogenous activators and inhibitors. Furthermore, we demonstrated that gain-of-function disease–associated mutations did not change PI(4,5)P_2_ sensitivity, indicating that the mutations do not increase channel activity *via* promoting PI(4,5)P_2_ activation. Our data provide mechanistic insights into regulation of TRPM3 by its key endogenous cofactor PI(4,5)P_2_.

## Results

**Figure 1:**
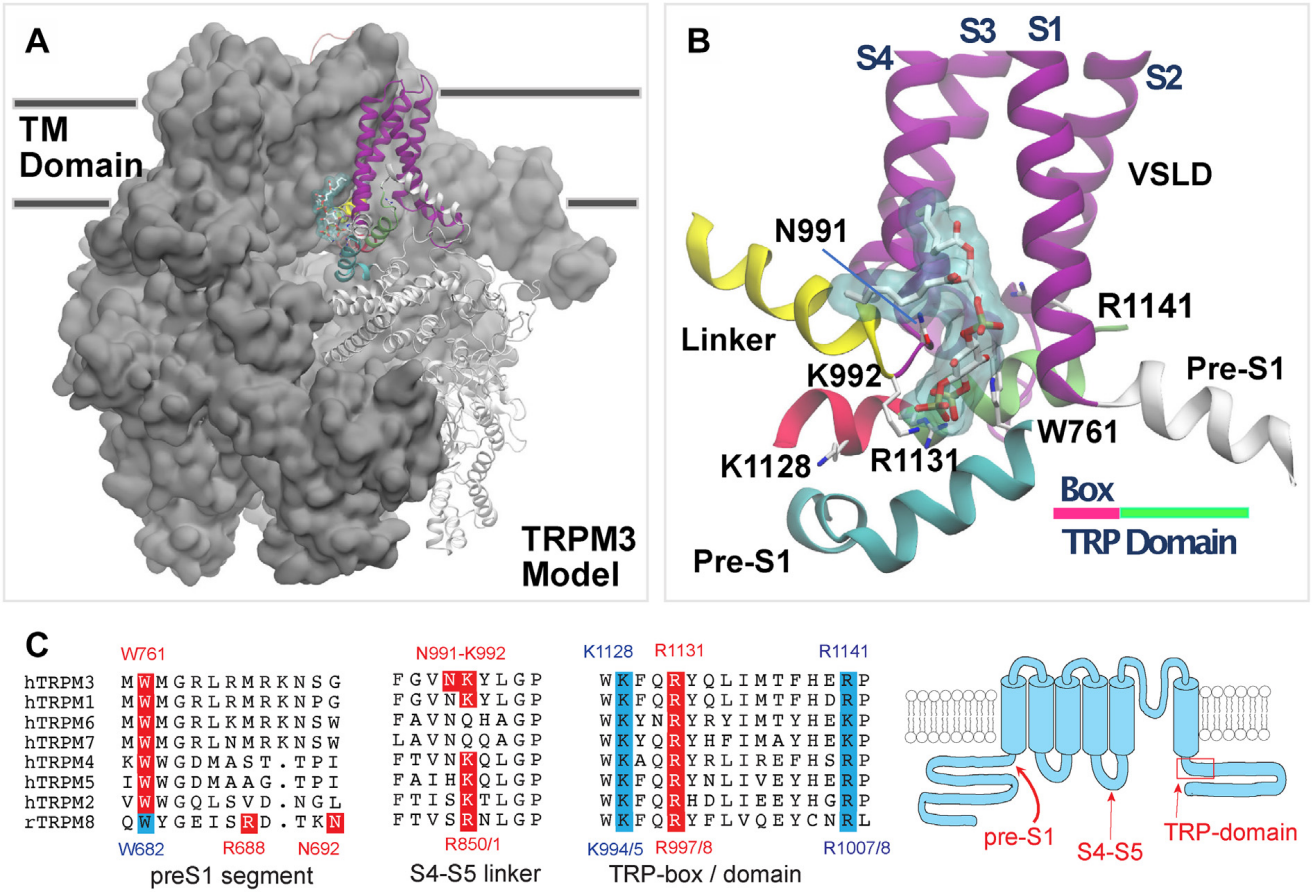
**Model of TRPM3 in complex with a PI(4,5)P**_**2**_**phospholipid.** The homology model of TRPM3 was built based on the structure of TRPM4 (PDB ID: 6BCJ) (29) as described in the Results and Experimental procedures sections. For visualization purposes, only one molecule of the phospholipid is shown. *A*, view from the transmembrane (TM) plane of TRPM3 tetramer. Protein atoms of three of the four protomers are shown in surface representation, colored in *gray*. In the fourth protomer, protein atoms are shown in new *cartoon* representation. Atoms in the pre-S1 domain (two separate ranges), voltage sensor–like domain (VSDL), and the S4–S5 linker are shown in new *cartoon* representation, colored in *cyan* and *white*, *magenta*, and *yellow*, respectively. The TRP domain is colored in *bright red* (TRP-box) and *green*. The remaining structural elements in the protomer are shown in *white* (*transparent*). The atoms of the phospholipid are shown in *licorice* representation, with C, N, and O atoms colored in *white*, *blue*, and *red*, respectively. For visualization purposes, only Cα and protein side-chain atoms are shown. Phospholipid atoms are also shown in surface representation, colored in *light blue* (*transparent*). In (*B*), close-up view of the PI(4,5)P_2_ binding site in TRPM3. Protein atoms are represented as *new cartoons*. Phospholipid atoms are represented as in (*A*). *C*, sequence alignment and *cartoon* of the PI(4,5)P_2_-interacting regions in TRPM3 and TRPM8. *Red* residues are in contact with PI(4,5)P_2_ in the TRPM8 cryo-EM structure(s) and/or in our TRPM3 model, when conserved in other TRPM channels, they are also labeled *red*. Residues in *cyan* in the TRP domain were experimentally characterized in this study or in Ref. (36). The location of the W682 residue in TRPM8 that we experimentally characterized is also noted here in *cyan*. The dual numbering S4–S5 loop and in the TRP domain indicates the difference in numbering between the rTRPM8 that we use in experiments and the fcTRPM8 used in cryo-EM studies. The MHR4 region in TRPM8 that contains the K605 PI(4,5)P_2_ contact residue in TRPM8 is not shown, as its sequence is not conserved in TRPM3. PDB, Protein Data Bank; PI(4,5)P_2_, phosphatidylinositol 4,5-bisphosphate; TRPM3, transient receptor potential melastatin 3.

Our goal in this study was to identify the PI(4,5)P_2_ binding site of TRPM3. As there is currently no experimentally determined TRPM3 structure available, we generated a homology model of the human TRPM3 (hTRPM3) based on the cryo-EM structure of the mouse TRPM4 in the apo state (Protein Data Bank [PDB] ID: 6BCJ) (29) (Fig. 1, *A* and *B*). The template was selected as the closest homolog to TRPM3 in the TRPM family with an experimental structure available when the model was built (see the Experimental procedures section and Scheme S1 for details). Our model of TRPM3 aligns very well with the models of TRPM3 from different organisms generated recently by AlphaFold (31, 32) (Fig. S1), as well as with a model of TRPM3 obtained using the experimental structure of mouse TRPM7 (33) in EDTA (PDB ID: 5ZX5) as the template (Fig. S2), all of which became available after our original homology model was built, providing *a posteriori* validation of our TRPM3 model.

**Figure 2:**
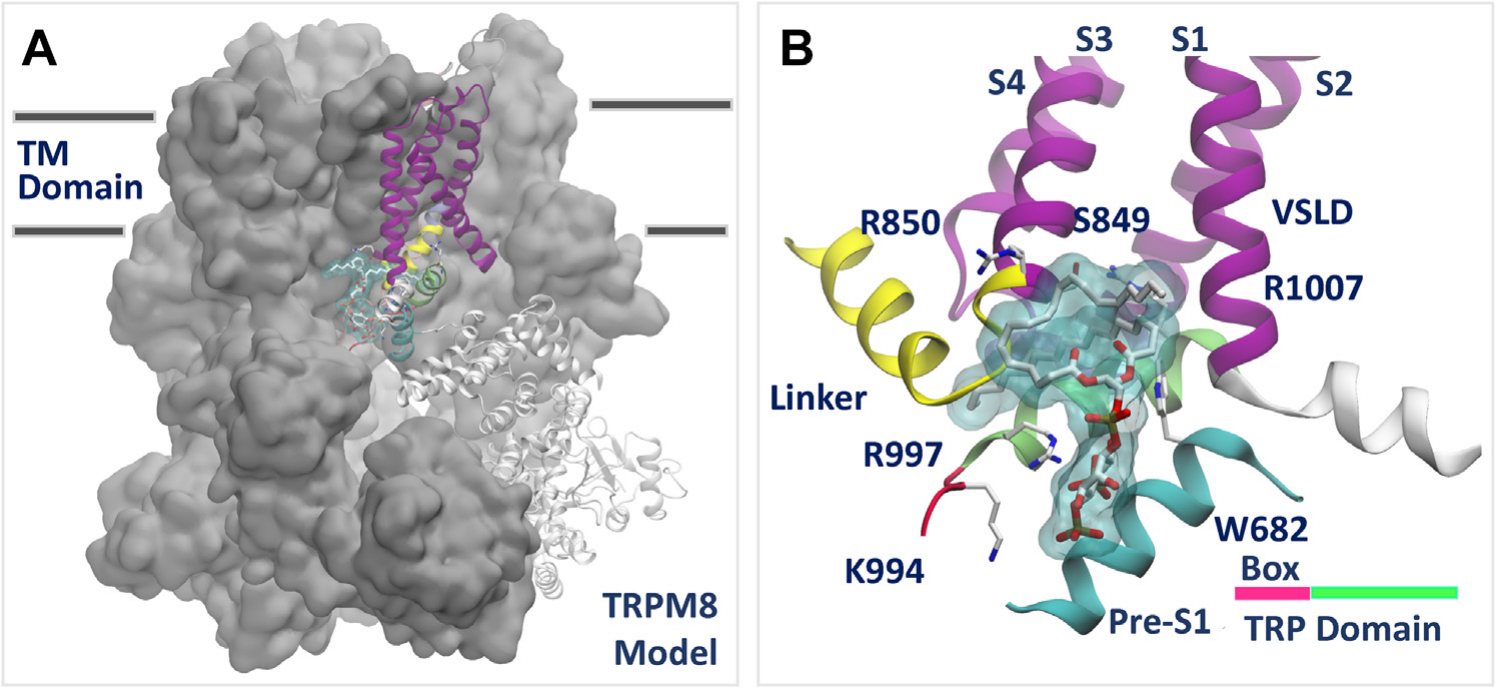
**Refined model of TRPM8 in complex with PI(4,5)P**_**2**_**.** PI(4,5)P_2_ was docked to the apo structure of the fcTRPM8 (PDB ID: 6BPQ) (30) as described in the Results and Experimental procedures sections. For visualization purposes, only one molecule of PI(4,5)P_2_ is shown. *A*, view from the transmembrane (TM) plane of TRPM8 tetramer. *B*, close-up view of the PI(4,5)P_2_ binding site in TRPM8. All representations are reproduced as for Figure 1. fcTRPM8, flycatcher apo TRPM8; PI(4,5)P_2_, phosphatidylinositol 4,5-bisphosphate; TRPM8, transient receptor potential melastatin 8.

Next, we identified putative residues interacting with PI(4,5)P_2_ in TRPM3 by using two complementary approaches. *First*, we scanned the surface of apo TRPM3 (model built on TRPM4) for putative binding sites using the program SiteMap (Schrödinger, LLC, 2018) (34, 35). *Second*, we relied on sequence and structural information available on TRPM8 to detect, by homology, which residues are likely to interact with PI(4,5)P_2_. Specifically, (1) we generated a sequence alignment of TRP-domain residues, K995, R998, and R1108, in the rat TRPM8, which are conserved among TRPM family members (Fig. 1*C*), and were previously suggested to play a key role in PI(4,5)P_2_ interactions (36) and (2) starting from the apo cryo-EM structure of the flycatcher apo TRPM8 (fcTRPM8) (PDB ID: 6BPQ) (30), we built a refined model of this channel bound to PI(4,5)P_2_ at a site that includes the conserved TRP-domain residues (Fig. 2, *A* and *B*). Comparing this complex with the apo-TRPM3 model showed that the most suitable site for lipid binding (*i.e.*, the top-scoring binding spot combining SiteMap predictions and structural information) in TRPM3 corresponded to the PI(4,5)P_2_ site identified in TRPM8. We used this lipid-binding site to generate a model of TRPM3 in complex with a version of PI(4,5)P_2_ with truncated tails (similar to the synthetic diC_8_ PI(4,5)P_2_, which is fully functional in experiments) by molecular docking using the program Glide (37). We ranked the lipid binding modes by the standard precision scoring function. The best binding mode in TRPM3, defined as the best docking score (kilocalorie/mole) obtained at the binding site similar to that in TRPM8, is shown in Figure 1, *A* and *B*.

The PI(4,5)P_2_ binding site in TRPM3 is formed by parts of the preS1 segment, the S4–S5 loop, and the proximal C-terminal TRP domain of the same subunit. The closest contact residues with PI(4,5)P_2_ are W761 in the preS1 segment, the N991 and K992 residues in segment connecting the voltage sensor–like domain (S1–S4) to the S4–S5 linker and the R1131 in the TRP domain (Fig. 1*B*). Figure 1*B* also shows the location of two additional residues, K1128 and R1141 in the TRP domain, which are not in close contact with PI(4,5)P_2_, but we experimentally characterized their mutations (see later). The numbering of these residues corresponds to the splice variant of hTRPM3 (hTRPM_1325_) (15, 38), which we used in the majority of our experiments.

Comparing our model of TRPM8 in complex with PI(4,5)P_2_ with the subsequently determined two cryo-EM structures of TRPM8 with PI(4,5)P_2_ (28), icilin (PDB ID: 6NR3), and with the menthol analog WS12 (PDB ID: 6NR2), offered *a posteriori* validation of our modeling (Table S1). Fig. S3 compares the PI(4,5)P_2_ binding pockets of the TRPM8–PI(4,5)P_2_–icilin structure, the TRPM8–PI(4,5)P_2_–WS12 structure, and our computational model. Our model superposes very well with both structures in the transmembrane domains, but it shows a better structural alignment of the PI(4,5)P_2_ binding site with the TRPM8–PI(4,5)P_2_–WS12 (PDB ID: 6NR2) structure than the TRPM8–PI(4,5)P_2_–icilin–calcium structure (PDB ID: 6NR3). In fact, the minimum RMSD values, calculated over amino-acid ranges facing the lipid-binding sites between any two aligned structures, were 1.49 and 2.34 Å, respectively (Table 1). Interestingly, the structural difference between the two experimental structures (minimum RMSD of 1.99 Å) is larger than that observed between our model and the closest experimental complex (minimum RMSD of 1.49 Å).

**Table 1:** Structural comparison between TRPM8 model and experimental structures

RMSD (Å)			
TRPM8 model	6NR2 (PI(4,5)P_2_ and WS12)	6NR3 (PI(4,5)P_2_, icilin, and Ca^2+^)	Selection
0	1.49	2.34	Sel-1
0	1.69	2.92	Sel-2
1.49	0	1.99	Sel-1
1.69	0	2.26	Sel-2
2.34	1.99	0.0	Sel-1
2.92	2.26	0.0	Sel-2

RMSD was calculated independently on two different TRPM8-residue selections (details are provided in Experimental procedures section) using the TRPM8 model or the TRPM8 experimental structures 6NR2 or 6NR3 as reference (RMSD = 0.0 Å).

Yin *et al.* (28) listed five key residues in their cryo-EM costructures critical for PI(4,5)P_2_ interaction: R997 in the TRP domain, R850 in the S4–S5 loop, N692 and R688 in the pre-S1 segment (Fig. 1*C*), and K605 in the neighboring N-terminal cytoplasmic Melastatin Homology Region 4 (MHR4) domain. All these residues, with the exception of R850, are in contact with, or very close to PI(4,5)P_2_ in our TRPM8–PI(4,5)P_2_ model. R850 is in contact with the acyl chain of PI(4,5)P_2_ in our model, and only in contact with the PI(4,5)P_2_ headgroup in the 6NR3, but not in the 6NR2 structure, which is consistent with the better alignment of our model with the 6NR2 PI(4,5)P_2_-TRPM8 structure. Overall, our TRPM8–PI(4,5)P_2_ docking model validates our computational approach to identify the TRPM3 PI(4,5)P_2_ binding site and suggests that PI(4,5)P_2_ likely binds to a site that is similar in TRPM3 and TRPM8.

Furthermore, superimposition of our model to the experimental structure of TRPM7 in EDTA (PDB ID: 5ZX5) (33) revealed that the docked PI(4,5)P_2_ in our model of TRPM3 fits well in a cavity of the experimental structure of TRPM7 that accommodates a detergent cholesteryl hemisuccinate molecule (Fig. S4). Whether this binding site is occupied by PI(4,5)P_2_ in TRPM7 in a cellular environment, remains to be determined, nevertheless the presence of this lipid-binding pocket in TRPM7 suggests that the location of the PI(4,5)P_2_ binding site may be conserved in multiple members of the TRPM subfamily.

In our TRPM3-PI(4,5)P_2_ model, residues K992 and R1131 are equivalent to the experimentally determined PI(4,5)P_2_ contact sites R850 and R997 in TRPM8, located in the S4–S5 loop and the TRP domain (Fig. 1*C*). Specifically, N991 is adjacent to K992, and W761 in the pre-S1 segment of TRPM3 is shifted six residues from the R688 residue in TRPM8. The equivalent of W761 in TRPM8 (W682) is relatively close to PI(4,5)P_2_ in TRPM8, and so is the equivalent of R688 in TRPM3 (M767) highlighting the generally similar importance of the pre-S1 segment in PI(4,5)P_2_ binding in the two channels. The largest difference between the two binding sites is that the MHR4 region, which carries K605 in TRPM8, is not conserved in TRPM3, and the equivalent residue is far away from PI(4,5)P_2_ in our TRPM3 model. Overall, the two channels bind PI(4,5)P_2_ in a similar, yet not identical manner (Fig. S5).

**Figure 3:**
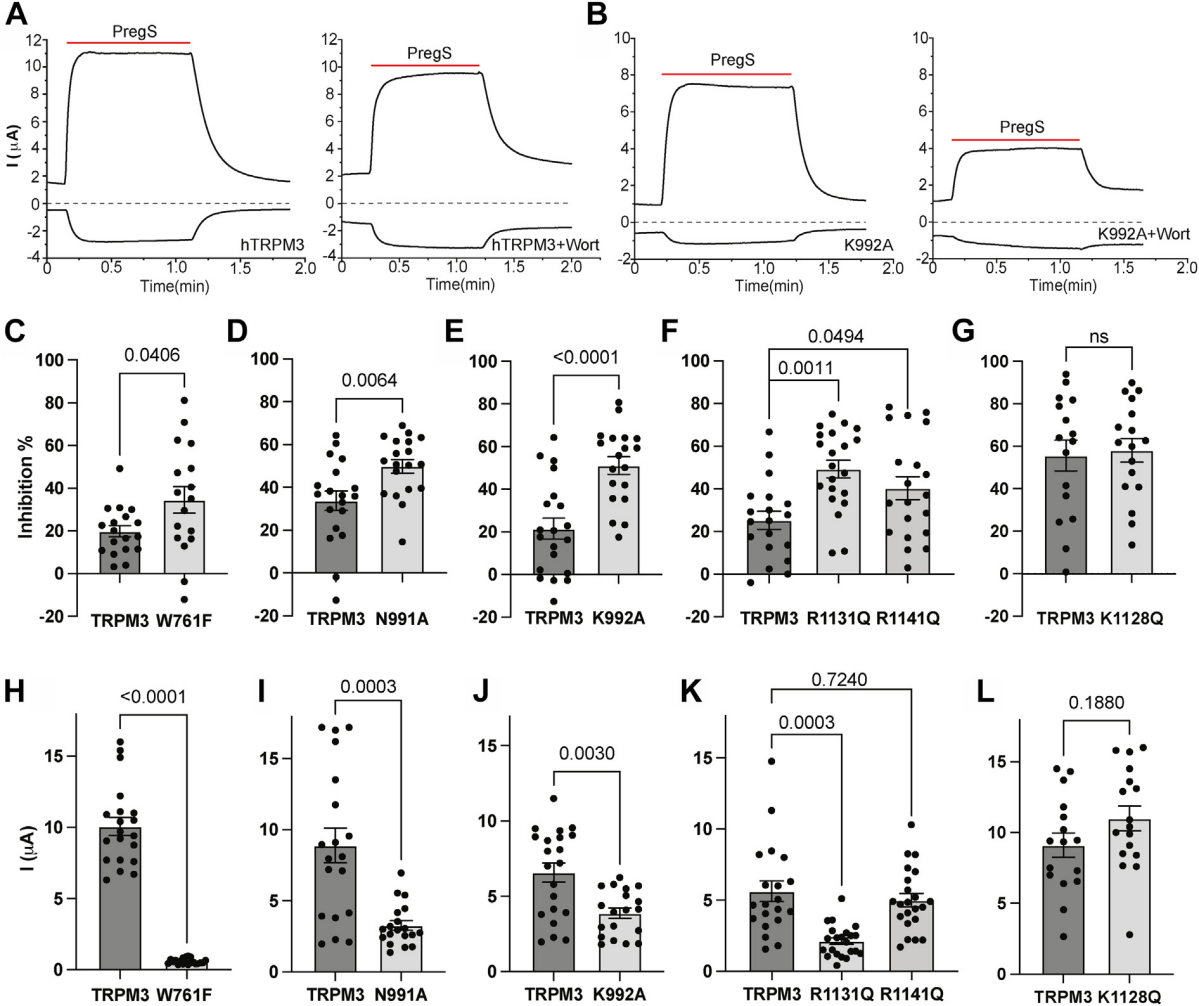
**Mutating putative PI(4,5)P**_**2**_**-interacting residues increases sensitivity of TRPM3 to inhibition by PI(4,5)P**_**2**_**depletion.** Human TRPM3_1325_ splice variant or its mutants were expressed in Xenopus oocytes, and two-electrode voltage-clamp (TEVC) experiments were performed to measure the activity of TRPM3 as described in the Experimental procedures section using a ramp protocol from −100 mV to +100 mV. Currents induced by 50 μM PregS were measured before and after treatment with 35 μM wortmannin to deplete PI(4,5)P_2_. *A* and *B*, representative traces of WT TRPM3 (*A*) and K992A mutant (*B*) before (*left*) and after (*right*) treatment of the same oocyte with 35 μM wortmannin for 2 h. *Top traces* show currents at +100 mV; *dash lines* indicate zero current; *bottom traces* show currents at −100 mV. Applications of 50 μM PregS are indicated by *red lines*. *C*–*G*, data summary of the percentage inhibition of PregS-induced currents by wortmannin treatment at 100 mV for different mutants: W761F (*C*), N991A (*D*), K992A (*E*), R1131Q and R1141Q (*F*), and K1128Q (*G*). *H*–*L*, current amplitudes of various mutants at 100 mV. Each symbol represents measurement of one oocyte from two independent preparations. Statistical significance was calculated with *t* test, or one-way ANOVA (*F* and *K*), *p* values are shown on *bar graphs*. For the overall ANOVA, *F* = 6.78, *p* = 0.0023 for panel *F*, and *F* = 13.01, *p* < 0.0001 for panel *K*. Individual panels show summary of measurements performed on the same experimental days. PI(4,5)P_2_, phosphatidylinositol 4,5-bisphosphate; PregS, pregnenolone sulfate; TRPM3, transient receptor potential melastatin 3.

Next, we mutated the predicted PI(4,5)P_2_-interacting residues in TRPM3 and tested the effects of the mutations on sensitivity to inhibition by decreasing PI(4,5)P_2_ levels. We expressed the WT and mutant channels in Xenopus oocytes and performed two-electrode voltage clamp (TEVC) experiments. We stimulated channel activity with 50 μM PregS and measured current amplitudes, then incubated the oocytes with 35 μM wortmannin for 2 h, and measured PregS-induced currents in the same oocytes (Fig. 3, *A* and *B*). Wortmannin at this concentration inhibits phosphatidylinositol 4-kinases and has been used to inhibit the activity of PI(4,5)P_2_-dependent ion channels (39). We showed earlier that at 35 nM, a concentration that selectively inhibits phosphoinositide 3-kinases (PI3K), wortmannin did not inhibit TRPM3 (21), indicating that TRPM3 inhibition by 35 μM wortmannin is caused by inhibition of phosphatidylinositol 4-kinase, not PI3K. Mutating a PI(4,5)P_2_-interacting residue is expected to increase inhibition by high concentrations of wortmannin (39). We mutated the TRP domain positively charged residues to Q, as equivalent mutations in TRPM8 were shown to be functional, and affect PI(4,5)P_2_ interactions (36). The rest of the residues we mutated to A, but the W761A mutant was nonfunctional, thus we functionally characterized W761F instead. Mutations of all computationally predicted PI(4,5)P_2_-interacting residues (W761F, N991A, K992A, and R1131Q) showed significantly higher inhibition after wortmannin treatment than WT TRPM3 (Fig. 3, *C–F*), and their current amplitudes were also significantly lower than WT TRPM3 (Fig. 3, *H–K*). We also generated two additional mutations in the TRP domain in residues that are not in contact with PI(4,5)P_2_, K1128Q and R1141Q. Both mutants showed similar current amplitudes to WT TRPM3 (Fig. 3, *K* and *L*). The K1128Q mutant showed similar inhibition to WT (Fig. 3*G*), but the R1141Q mutant showed a small but significant increase in wortmannin inhibition compared with WT (Fig. 3*F*). This mutation is equivalent to R1008Q in the rat TRPM8, which reduced both PI(4,5)P_2_ and menthol sensitivity (36), but it was in contact with the menthol analog WS12, but not with PI(4,5)P_2_ in the cryo-EM structure of fcTRPM8 (R1007) (28). Therefore, it is possible that this mutation affected PI(4,5)P_2_ sensitivity indirectly.

Next, we confirmed our data in whole-cell patch-clamp experiments using the rapamycin-inducible 4′ 5′ phosphoinositide phosphatase pseudojanin (40). Fig. S6, *A–C* shows that 100 nM rapamycin induced a significantly higher inhibition of the N993A mutant of the mouse TRPM3α2 (mTRPM3α2; equivalent of N991A in hTRPM3) than the WT mTRPM3α2 when the channels were stimulated with 25 μM PregS. Next, we stimulated the mTRPM3α2 with the combination of 25 μM PregS and 10 μM clotrimazole, which was shown to open an alternative pore, characterized by larger currents and less prominent outward rectification (41). Application of 100 nM rapamycin induced a significantly larger inhibition of currents induced by clotrimazole plus PregS in the N993A mutant compared with the WT mTRPM3α2 (Fig. S6, *E–G*). Current amplitudes for the N993A mutant were also lower than those in the WT TRPM3 (Fig. S6, *D* and *H*).

Mutation of the PI(4,5)P_2_ contact site R998Q resulted in a right shift in the diC_8_ PI(4,5)P_2_ dose response in excised patches (36). TRPM3 currents in excised patches show a less steep concentration dependence, with no clear saturation at higher concentrations (21). This and the low current amplitudes in the mutants prevented us from reliably comparing PI(4,5)P_2_ dose responses in our mutants. It was reported for TRPV1 that mutating a putative PI(4,5)P_2_-interacting residue increased the relative efficiency of PI(4)P to stimulate channel activity compared with PI(4,5)P_2_ (42). Therefore, we tested the relative effects of PI(4)P and PI(4,5)P_2_ on the N991A mutant. Fig. S7, *A–C* shows that the relative effect of PI(4)P compared with PI(4,5)P_2_ did not change.

**Figure 4:**
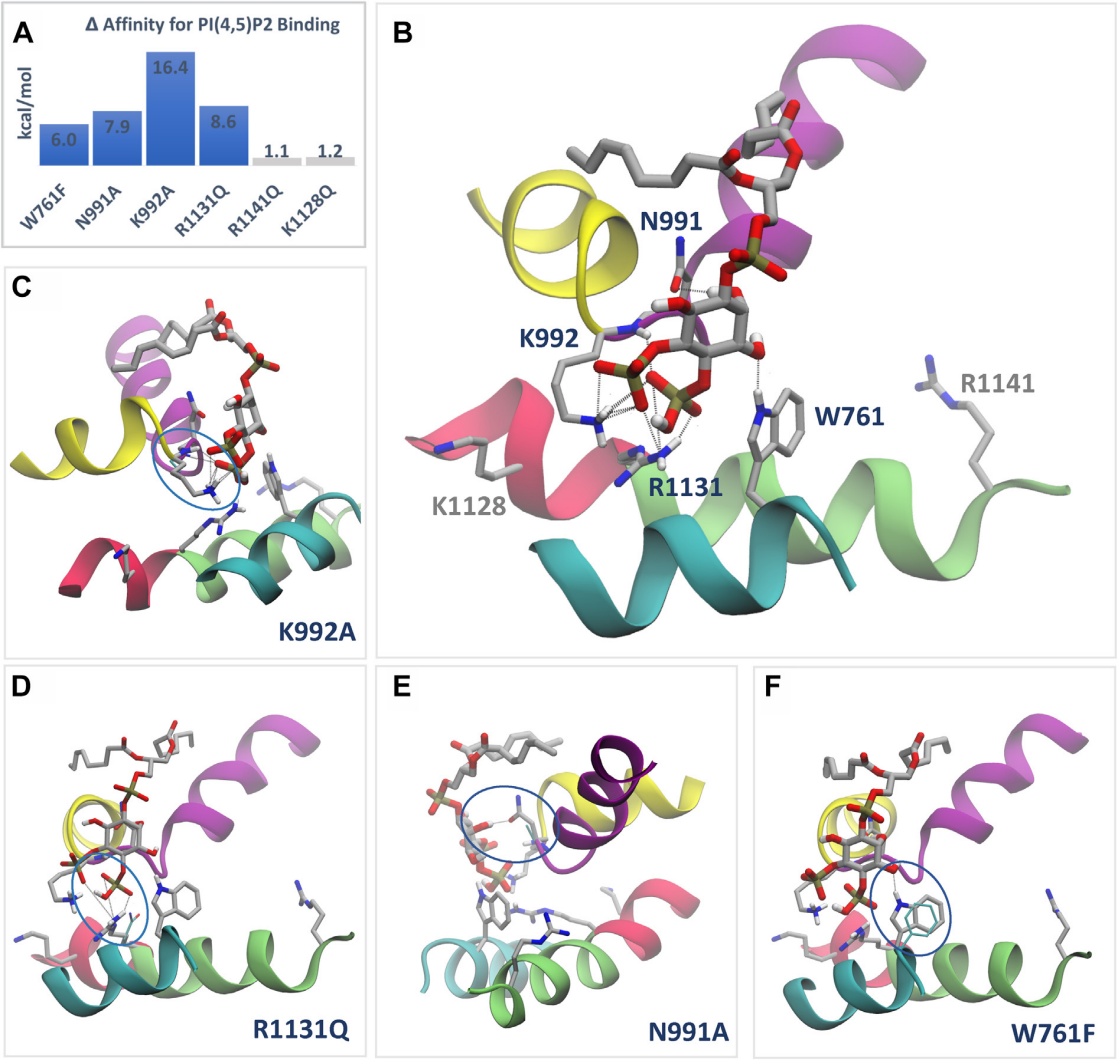
**Effect of mutations in the PI(4,5)P**_**2**_**binding site residues of TRPM3.** Binding affinity changes were calculated *via* the MM/GBSA method, as described in the Experimental procedures section. *A*, change in binding affinity (ΔΔG; kcal/mol) upon mutating protein residues *in silico* in the PI(4,5)P_2_ binding site of TRPM3. In *blue*, mutants that bind significantly worse than the native protein, indicating loss of interaction with PI(4,5)P_2_ upon mutations. In *gray*, mutants with no significant effect on binding. *B*–*F*, binding mode of PI(4,5)P_2_ to native and mutant TRPM3 channels. In (*B*), native TRPM3. In (*C*–*F*), for any mutant with significant loss of interaction with the phospholipid (in *blue color* in *A*), the effect of each individual mutation is shown by visualizing the mutated residue superposed to the native counterpart; the loss of any hydrogen bond or salt bridge interactions with the phospholipid is highlighted in *oval shapes*. Protein atoms are shown in new *cartoon* representation, in *gray* and *cyan* color for native and mutant channels, respectively. For visualization purposes, hydrogens not engaged in interactions with the protein–ligand interactions are *hidden*. Ligand atoms are in *licorice* representation, with C, O, H, and P atoms colored in *gray*, *red*, *white*, and *yellow*, respectively. Hydrogen bonds and salt bridges are represented as *dotted lines*. MM/GBSA, molecular mechanics/generalized Born surface area; PI(4,5)P_2_, phosphatidylinositol 4,5-bisphosphate; TRPM3, transient receptor potential melastatin 3.

Next, we used MM/GBSA calculations (Fig. 4) to predict the changes in the binding free energy (ΔΔG) of PI(4,5)P_2_ to the native (WT) TRPM3 *versus* the mutant channels that were characterized in Figure 3. In particular, we used the VSGB 2.0 model (43), in which the solvation free energy is approximated with an optimized model based on the surface generalized Born method (44) and the variable dielectric treatment of polarization (45) for protein residues. We note that we did not include an implicit membrane model (*i.e.*, a low-dielectric slab) and, therefore, the results should be taken as a qualitative indication.

As shown in Figure 4, the binding of PI(4,5)P_2_ is guided by a number of stabilizing interactions (Fig. 4*A*) established with key contact residues (Fig. 4, *B–F* and Table S2). Mutations of all these residues in our model resulted in a decreased PI(4,5)P_2_ binding affinity (for a native protein to bind better than the mutant, the calculated ΔΔG value is positive). Specifically, K992A had a more prominent effect than R1131Q, N991A, and W761F, respectively. This correlates well with K992A also having the most pronounced effect on inhibition by PI(4,5)P_2_ depletion (Fig. 3*E*). Regarding the binding modes, K992 engages in multiple interactions with PI(4,5)P_2_, including three hydrogen bonds and three salt bridges. Mutating K992 to alanine resulted in the loss of all these interactions with the exception of one hydrogen bond, the only interaction established by the amino acid backbone (Fig. 4*C*). Similar behavior was observed with the mutation R1131Q, the contact residue exerting the second largest effect on the binding affinity, which resulted in the loss of one hydrogen bond and two salt bridges (Fig. 4*D*), all established by the residue side chain. Next, mutating N991 to alanine and W761 to phenylalanine resulted in the loss of one hydrogen bond each (Fig. 4, *E* and *F*, respectively). To further corroborate our results, we performed additional sets of calculations of the binding affinity change upon mutation (Table S2 and Fig. S8) using, as the starting configurations, the docking poses of PI(4,5)P_2_ with even shorter tails than the ones included in the model, and with headgroups featuring different protonation states (hereinafter referred to as shortest-PI(4,5)P_2_). These calculations are clearly reproducible and agree with experimental observations. Although the trend is maintained overall (Figs. 4*A* and S8), W761F shows a reduction in the binding affinity change for shortest-PI(4,5)P_2_ (Fig. S8, *light blue*), because of headgroup protonation states that prevent interactions *via* hydrogen-bond formation. Hence, it appears from our calculations, that the PI(4,5)P_2_ protonation state featured in the proposed TRPM3 model (Fig. 4) is the one that favorably affects the binding of the phospholipid to the native protein. Interestingly, the protonation state of PI(4,5)P_2_ was suggested to critically impact the binding to related TRP channels (46). Furthermore, among the mutations leading to a decrease of binding affinity, W761 is located the furthest from PI(4,5)P_2_ (see structural model), and therefore, it is not unexpected that mutating this residue could affect to a lesser extent the binding of a smaller ligand (Table S2).

Of the remaining two mutations (Fig. 4, *A* and *B*), R1128Q had only a very small effect on both ΔΔG and the related binding mode, which correlates well with it not being in close contact with PI(4,5)P_2_ in our model, and the lack of effect on wortmannin inhibition. The R1141Q mutant, which is also not a PI(4,5)P_2_ contact site, also had only a minimal effect on both ΔΔG and the related binding mode, indicating that the small, but significant, effect on wortmannin inhibition was likely because of indirect effects. Overall, all Δ affinity calculations supported our computational docking and agreed with the experimental functional characterization of the PI(4,5)P_2_-interacting residues.

It is worth mentioning that, although our binding model likely captures a highly represented conformational state sampled by the TRPM3 channel when bound to PI(4,5)P_2_, it is expected that other states may exist featuring alternative networks of interactions yet compatible with the proposed phospholipids-binding site model.

**Figure 5:**
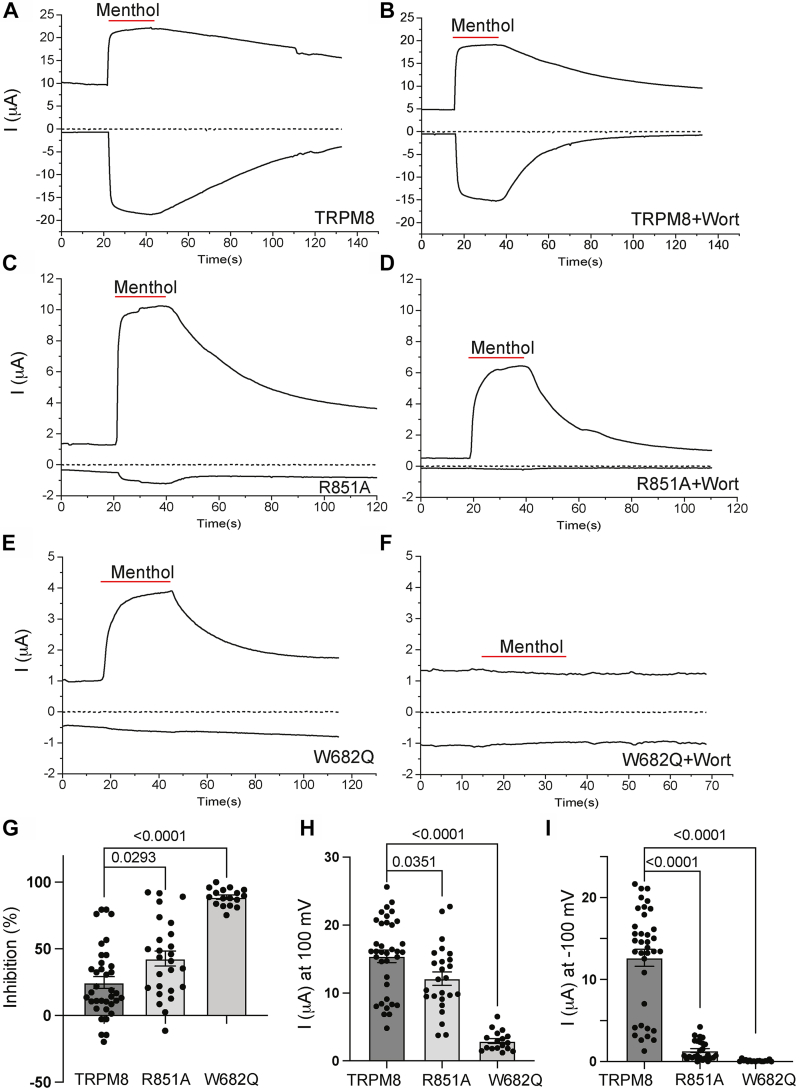
**Mutating putative PI(4,5)P**_**2**_**-interacting residues increases sensitivity of TRPM8 to inhibition by PI(4,5)P**_**2**_**depletion.** Rat TRPM8 or its mutants were expressed in Xenopus oocytes. TEVC experiments were performed as described in the Experimental procedures section using ramp protocol from −100 mV to +100 mV. Menthol (500 μM) was applied to activate TRPM8 channels, and 35 μM wortmannin was applied for 2 h to deplete PI(4,5)P_2_. *A*–*F*, representative traces of TRPM8 before (*A*) and after wortmannin treatment (*B*), R851A before (*C*) and after wortmannin treatment (*D*) and W682Q before (*E*) and after wortmannin treatment (*F*). *Top traces* show currents at 100 mV; *dash lines* indicate 0 current; *bottom traces* show currents at −100 mV. Applications of 500 μM menthol are indicated by *red lines*. *G*, summary of inhibition evoked wortmannin treatment at 100 mV (basal plus menthol-induced current after leak subtraction) plotted for WT, R851A, and W682Q. *H* and *I*, current amplitudes of all three groups at 100 mV (*H*) and −100 mV (*I*). Each *symbol* represents an individual oocyte. All experiments were from two to three independent preparations. Statistical significance was calculated with one-way ANOVA. *p* Values for individual comparisons are shown on *bar graphs*. For the overall ANOVA, *F* = 49.08, *p* < 0.0001 for panel *G*, and *F* = 53.25, *p* < 0.0001 for panel *H*, and *F* = 122.7, *p* < 0.0001 for panel *I*. PI(4,5)P_2_, phosphatidylinositol 4,5-bisphosphate; TEVC, two-electrode voltage clamp; TRPM3, transient receptor potential melastatin 8.

Next, we mutated two residues in the rTRPM8 that are equivalent to PI(4,5)P_2_-interacting residues in our TRPM3 model. The R851 residue in TRPM8 corresponds to the K992 residue in the S4–S5 linker in TRPM3 (Fig. 1*C*), and it was in direct contact with PI(4,5)P_2_ in the cryo-EM structure of the fcTRPM8 (R850) (28). The W682 residue is the equivalent of W761 in TRPM3 (Fig. 1*C*), and while is not in a direct contact with PI(4,5)P_2_ in the fcTRPM8 cryo-EM structure (R850), it is located relatively close. Since the W682A mutant was nonfunctional, we characterized the W682Q, which displayed small, yet measurable, menthol-induced currents. Figure 5, *A–G* shows that both the R851Q and the W682Q mutants showed significantly higher level of inhibition by wortmannin, with W682Q having a larger effect. Current amplitudes showed a similar pattern; both mutants were significantly lower than WT TRPM8, and the W682Q having a larger effect (Fig. 5*H*). The decrease in amplitudes was even more pronounced at negative voltages for inward currents (Fig. 5*I*), in agreement with earlier results with the R995Q PI(4,5)P_2_ mutant (36). This is likely caused by the allosteric interaction between PI(4,5)P_2_ and voltage in modulating TRPM8. Wortmannin treatment substantially accelerated deactivation after cessation of menthol stimulation (Fig. 5, *A–D*), which is in contrast to TRPM3, where the deactivation kinetics after washing out PregS was not affected by wortmannin (Fig. 3, *A* and *B*).

**Figure 6:**
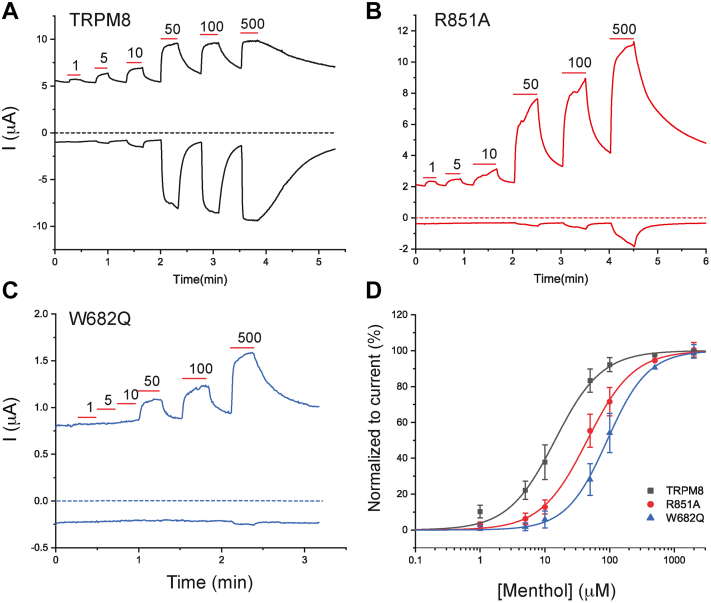
**Mutating putative PI(4,5)P**_**2**_**-interacting residues decreases sensitivity of TRPM8 to menthol activation.** TEVC experiments were performed using a ramp protocol from −100 mV to 100 mV, as described in the Experimental procedures section. *A*–*C*, representative traces of TRPM8 (*A*), R851A (*B*), and W682Q (*C*). *Top traces* show currents at +100 mV; *dash lines* indicate zero current; *bottom traces* show currents at −100 mV. Applications of various concentrations of menthol (μM) are indicated by *red lines*. *D*, Hill1 fit of the concentration dependence of menthol at 100 mV for TRPM8 and mutated channels. Currents were initially normalized to the current evoked by 500 μM menthol, then renormalized to the maximum current from the hill fits for plotting. Symbols represent mean ± SD for n = 12 to 13 measurements from two different oocyte preparations. PI(4,5)P_2_, phosphatidylinositol 4,5-bisphosphate; TEVC, two-electrode voltage clamp; TRPM8, transient receptor potential melastatin 8.

Stimulation with menthol, or cold, was shown to increase the apparent affinity of TRPM8 for PI(4,5)P_2_ (36) indicating an allosteric interaction between menthol and PI(4,5)P_2_ activation. Next, we asked if this allosteric interaction also happens in the opposite direction and tested if mutations of the PI(4,5)P_2-_interacting residues in TRPM8 have an effect on agonist sensitivity. Figure 6, *A–D* shows that both the R851A and the W682Q mutant had right shifted menthol dose response. Similar to the effect on current amplitudes and wortmannin inhibition, the effect of the W682Q mutant (Fig. 6, *C* and *D*) was more pronounced than that of R851A (Fig. 6, *B* and *D*).

**Figure 7:**
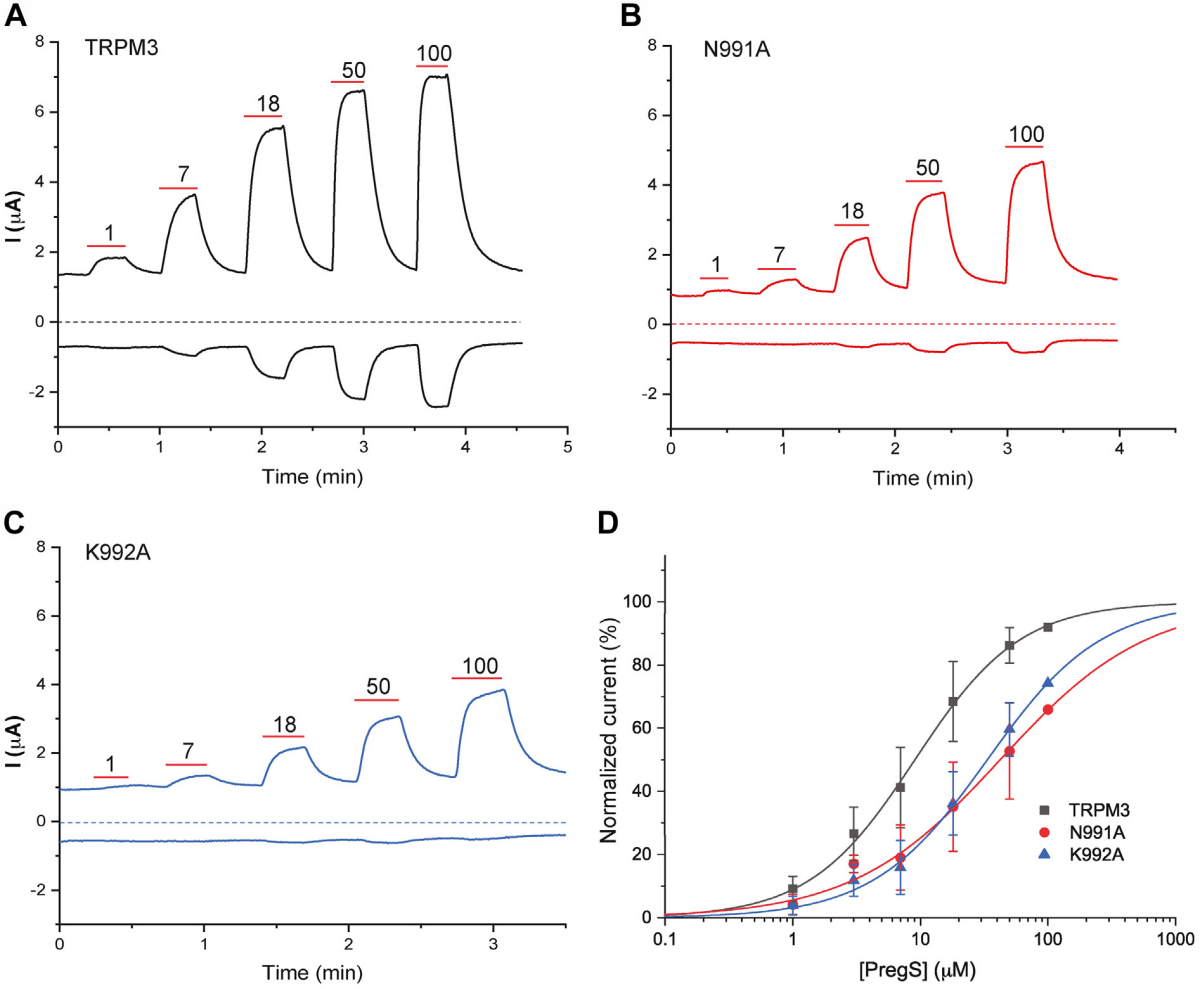
**Mutating putative PI(4,5)P**_**2**_**-interacting residues decreases sensitivity of TRPM3 to PregS activation.** RNAs coding for either the TRPM3_1325_ splice variant or its mutants were injected into Xenopus oocytes. TEVC was performed to measure TRPM3 currents using a ramp protocol from −100 mV to 100 mV, as described in the Experimental procedures section. *A*–*C*, representative traces of hTRPM3 WT (*A*), N991A (*B*), and K992A (*C*). *Top traces* show currents at +100 mV; *dash lines* indicate zero current; *bottom traces* show currents at −100 mV. Applications of various concentrations of PregS (micromolar) are indicated by *red lines*. *D*, Hill1 fit of the concentration dependence of PregS at 100 mV for TRPM3 and mutated channels. Currents were initially normalized to the current evoked by 100 μM PregS and then renormalized to the maximum current from the hill fits for plotting. *Symbols* represent mean ± SD for n = 12 to 13 measurements from two different oocyte preparations. hTRPM3, human TRPM3; PI(4,5)P_2_, phosphatidylinositol 4,5-bisphosphate; PregS, pregnenolone sulfate; TEVC, two-electrode voltage clamp; TRPM3, transient receptor potential melastatin 3.

We also tested if a similar allosteric effect also exists in TRPM3. Figure 7, *A*–*D* shows that both the N991A and the K992A mutant shifted the PregS dose response to the right.

**Figure 8:**
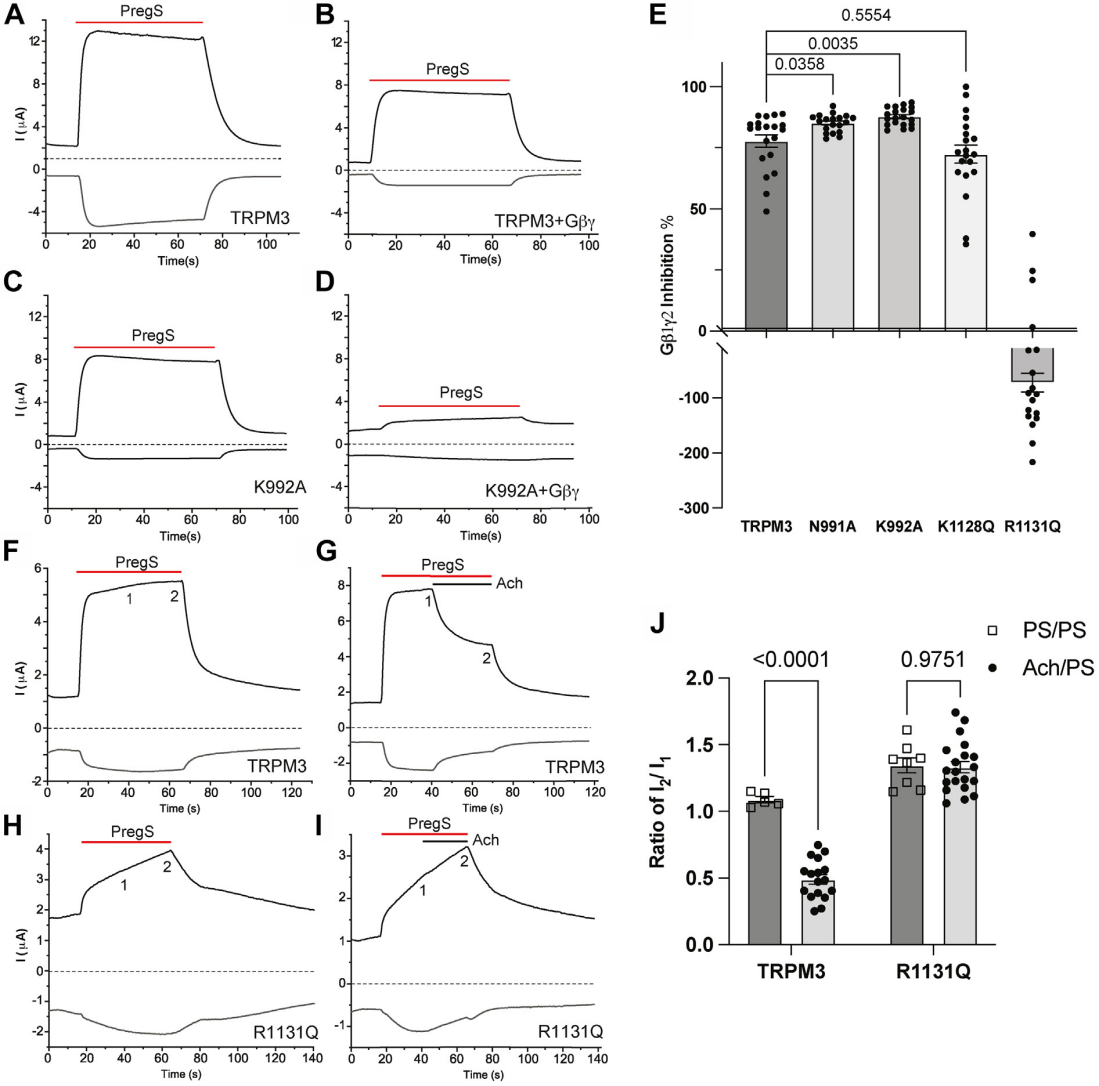
**Relationship between Gβγ and PI(4,5)P**_**2**_**regulation of TRPM3.** hTRPM3 or its mutants were expressed in oocytes with or without Gβ1γ2 subunits for *A*–*E* or with muscarinic hM2 receptors for *F*–*J*. TEVC was used to measure channel activity using a ramp protocol from −100 mV to 100 mV as described in the Experimental procedures section. *A*–*D*, representative traces of TRPM3 (*A*), TRPM3 coexpressed with Gβγ subunits (*B*), K992A (*C*) and K992A coexpressed with Gβγ subunits (*D*). *E*, data summary shows the inhibition caused by Gβγ subunits in different mutant groups. Percentages of inhibition were calculated by normalizing decreased current amplitudes to the average currents induced by 50 μM PregS in control oocytes without Gβγ subunits. *F*–*J*, representative traces of TRPM3 treated with PregS alone (*F*), TRPM3 treated with PregS and acetylcholine (*G*), R1131Q treated with PregS alone (*H*), and TRPM3 treated with PregS and acetylcholine (*I*). *Top traces* show currents at +100 mV; *dash lines* indicate zero current; *bottom traces* show currents at −100 mV. Application of 50 μM PregS is indicated by *red lines*, and application of 5 μM acetylcholine is indicated by *black lines*. PregS-induced currents in the R1131Q mutant channels showed a continuous increase after application of PregS, which necessitated comparison to control cells where PregS was applied for the same length of time without the application of ACh (*H*). *J*, data summary shows the ratio of current amplitudes between points 2 and 1 indicated on the representative traces. Each *symbol* represents an individual oocyte from three (*E*) and two (*J*) independent preparations. Statistical significance was calculated with one-way ANOVA (*E*) (*F* = 9.025, *p* = 0.001) and two-way ANOVA (*J*) (*F* = 29.03 and *p* < 0.0001) for interaction between ACh and mutation. *p* Values for post hoc individual comparisons are shown on *bar graphs*. ACh, acetylcholine; hM2, human M2 muscarinic receptor; hTRPM3, human TRPM3; PI(4,5)P_2,_ phosphatidylinositol 4,5-bisphosphate; PregS, pregnenolone sulfate; TEVC, two-electrode voltage clamp; TRPM3, transient receptor potential melastatin 3.

TRPM3 activity is inhibited by direct binding of Gβγ to the channel (9). To test if an allosteric interaction between Gβγ inhibition and PI(4,5)P_2_ activation is present, we expressed WT and mutant TRPM3 channels with or without Gβ1γ2 in Xenopus oocytes and measured PregS-induced currents. The N991A and K992A mutants were inhibited significantly more by Gβγ than WT TRPM3 (Fig. 8, *A–E*). The K1128Q mutant, which did not affect PI(4,5)P_2_ sensitivity, had no effect on Gβγ inhibition either (Fig. 8*E*). Interestingly, the R1131Q mutant was not inhibited, rather potentiated by coexpressing Gβγ (Fig. 8*E*). Consistently with the lack of inhibition by Gβγ, the R1131Q mutant was also not inhibited by stimulating Gi-coupled M2 muscarinic acetylcholine receptors (Fig. 8, *F–J*). These data indicate that while there is an allosteric interaction between PI(4,5)P_2_ and Gβγ, the R1131 residue in the TRP domain also plays some role in transmitting the inhibitory effect of Gβγ.

**Figure 9:**
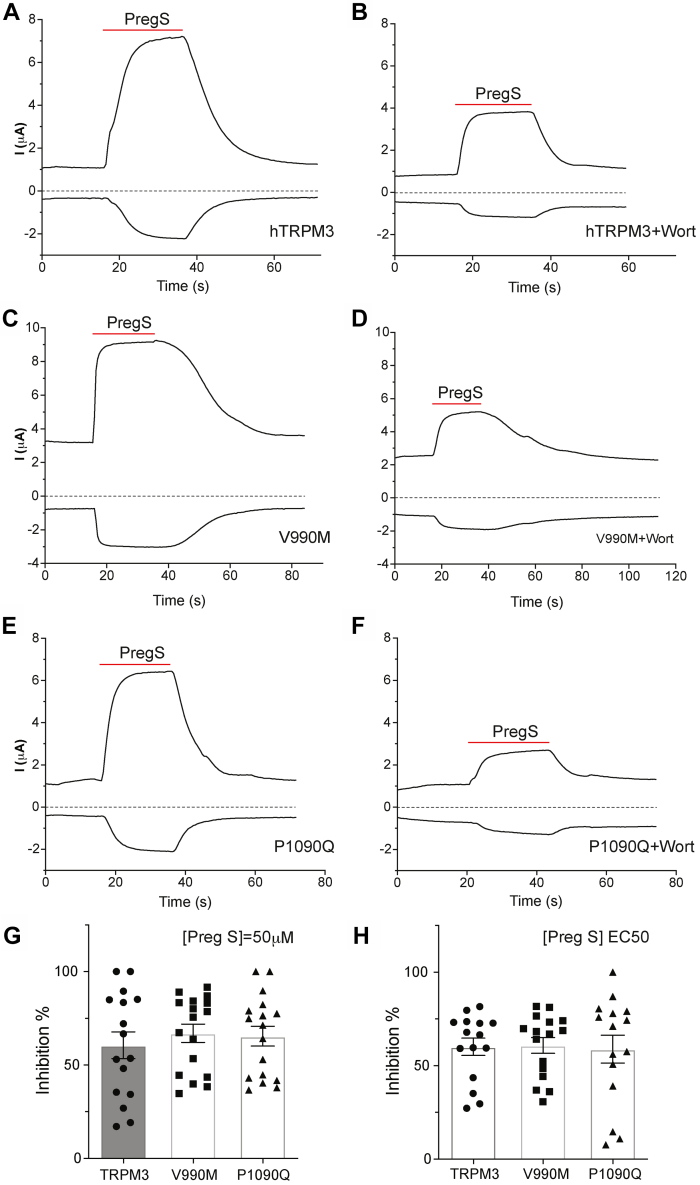
**Disease-associated gain-of-function mutants do not change the TRPM3 sensitivity to the PI(4,5)P**_**2**_**depletion.** hTRPM3, V990M, or P1090Q was expressed in oocytes, and 35 μM wortmannin (2 h) was used to deplete PI(4,5)P_2_. TEVC was performed as described in the Experimental procedures section. *A*–*F*, representative traces of hTRPM3 (*A*), hTRPM3 after wortmannin treatment (*B*), V990M (*C*), V990M after wortmannin treatment (*D*), P1090Q (*E*) and P1090Q after wortmannin treatment (*F*). *Top traces* show currents at +100 mV; *dash lines* indicate zero current; *bottom traces* show currents at −100 mV. Application of 50 μM PregS is indicated by *red lines*. *G*, data summary of wortmannin inhibition of currents induced by 50 μM PregS. *H*, data summary of wortmannin inhibition of currents induced by EC_50_ concentrations of PregS, 17 μM, 0.6 μM, and 7 μM for hTRPM3, V990M, and P1090Q, respectively. *Symbols* represent individual oocytes from three (*G*) and two (*H*) different preparations. hTRPM3, human TRPM3; PI(4,5)P_2_, phosphatidylinositol 4,5-bisphosphate; PregS, pregnenolone sulfate; TEVC, two-electrode voltage clamp; TRPM3, transient receptor potential melastatin 3.

Gain-of-function mutations in TRPM3 have recently been shown to cause intellectual disability and seizures (13, 14, 15). The two disease-associated mutations, V990M and P1090Q, were shown to increase basal channel activity, as well as increase in agonist sensitivity and increase in heat sensitivity, with V990M affecting agonist sensitivity more prominently, whereas P1090Q predominantly affecting heat sensitivity (15). Next, we tested if the increased basal activity and agonist sensitivity also translated into higher sensitivity to PI(4,5)P_2_. When WT (Fig. 9, *A* and *B*) and V990M (Fig. 9, *C* and *D*) and P1090Q mutant channels (Fig. 9, *E* and *F*) were treated with 35 μM wortmannin for 2 h, currents evoked by 50 μM PregS were inhibited to a similar extent (Fig. 9*G*). PregS-induced average current amplitudes were not significantly different in the mutant and WT channels (not shown), similar to our earlier data (15), presumably because the overactive channels tend to damage the cells expressing them and thus in the surviving oocytes are selected for lower expression levels of the mutants. The mutants were also inhibited to a similar extent to WT channels by wortmannin when currents were evoked by PregS corresponding to the respective EC_50_ (15) of the mutant and WT channels (Fig. 9*H*). These data indicate that the disease mutants do not increase channel activity by increasing their apparent affinity for PI(4,5)P_2_.

## Discussion

Our work aims to understand the molecular mechanism of PI(4,5)P_2_ regulation of TRPM3. We used computational docking, changes in the binding affinity estimated by computational mutagenesis, site-directed mutagenesis, and electrophysiology to identify PI(4,5)P_2_-interacting residues in the channel protein. Our data indicate that residues in three regions, the pre-S1 segment, the S4–S5 loop, and the TRP domain, play important roles in forming the PI(4,5)P_2_ binding site in TRPM3. Mutations of PI(4,5)P_2-_interacting residues decreased the binding affinity *in silico* (positive ΔΔG values in Figure 4) indicating that the native protein binds better than the mutant) and increased sensitivity to inhibition by decreasing PI(4,5)P_2_ levels in electrophysiology experiments (Fig. 3). Mutating PI(4,5)P_2_-interacting residues also decreased sensitivity to PregS activation and increased sensitivity to Gβγ inhibition indicating allosteric interaction between PI(4,5)P_2_ and agonists as well as a physiological inhibitor. On the other hand, disease-associated gain-of-function mutations did not change PI(4,5)P_2_ sensitivity, indicating that the mutations did not increase channel activity by enhancing PI(4,5)P_2_ activation.

There are currently five channels in the TRPM family for which structural data are available (47): TRPM2 (48), TRPM4 (49), TRPM5 (50), TRPM7 (33), and TRPM8 (28). While all these channels have been shown to be positively regulated by PI(4,5)P_2_ (23), only TRPM8 has a costructure with this lipid (28). When we compare the PI(4,5)P_2_ binding site in TRPM8 revealed by the structural study with our computationally identified binding site in TRPM3, the two overlap, sharing some of the interacting residues, with some differences (Figs. 1*C* and S5). Overall, the preS1 segment, the S4–S5 loop, and the TRP domain are involved in both channels in forming the PI(4,5)P_2_ binding site. The R1131 residue in the TRP domain in TRPM3 is equivalent to the R997 PI(4,5)P_2_ contact residue in the fcTRPM8 structure (28), and to the R998 residue in the rat TRPM8, which was proposed as a PI(4,5)P_2_-interacting residue and experimentally shown to exhibit decreased PI(4,5)P_2_ sensitivity before structures became available (36). The K992 residue in the S4–S5 loop of TRPM3 is equivalent to the R850 PI(4,5)P_2_ contact residue in the fcTRPM8 structure and to the R851 residue in the rat TRPM8 that we characterized in this study (Figs. 5 and 6). The pre-S1 segment of the fcTRPM8 has two PI(4,5)P_2_ contact residues R688 and N692 (Fig. 1*C*). These residues are not conserved in TRPM3 (Fig. 1*C*); yet the equivalent residues in our TRPM3 model, that is, M767 and G672, are both located within 5 Å of the PI(4,5)P_2_ headgroup (Fig. S5*D*), with M767 engaging hydrophobic interactions that stabilize the overall complex. The W761 PI(4,5)P_2_ contact residue in the pre-S1 of TRPM3, is equivalent to the W682 residue in TRPM8, which is close to the R688 residue as well as to PI(4,5)P_2_, but it was not close enough to designate it as a PI(4,5)P_2_ contact site in TRPM8 (28). Interestingly, when we mutated this residue to a glutamine (W682Q) in the rat TRPM8, it behaved similar to the W761F mutation in TRPM3, that is, it increased sensitivity to PI(4,5)P_2_ depletion (Fig. 5). Whether this residue is in a closer contact with PI(4,5)P_2_ in a cellular environment in the rat TRPM8, or its mutation affected PI(4,5)P_2_ interactions indirectly, or both, it is difficult to tell. Finally, the K605 residue from an adjacent cytoplasmic MHR4 domain was also in contact with PI(4,5)P_2_ in TRPM8. This residue is not conserved in TRPM3 and was not close to PI(4,5)P_2_ in our homology model.

It is well established that channel agonists can increase PI(4,5)P_2_ sensitivity (apparent affinity) for various PI(4,5)P_2_-sensitive ion channels. For example, the apparent affinity of the G protein–activated inwardly rectifying K^+^ channel GIRK4 (Kir3.4) for PI(4,5)P_2_ is increased by factors that stimulate channel activity, such as Gβγ and intracellular Na^+^ (51). The apparent affinity of TRPM8 for PI(4,5)P_2_ was shown to be increased by both cold and menthol (36), and the apparent affinity of TRPV1 for PI(4,5)P_2_ activation was increased by capsaicin (52). The opposite was also proposed, as a mutation in the putative PI(4,5)P_2_-interacting residue R1008 in TRPM8 not only decreased apparent affinity for PI(4,5)P_2_ but also induced a marked right shift in the menthol dose response (36). In the view of the structures of TRPM8 however, this residue is likely to be a menthol-interacting residue, as it was in close contact in the TRPM8 structure with the menthol analog WS12, but not with PI(4,5)P_2_ (28), therefore, it most likely primarily affected menthol sensitivity, and the effect on PI(4,5)P_2_ was a secondary allosteric effect. Our data indicate that in both TRPM8 and TRPM3, mutating PI(4,5)P_2_ contact residues also decrease agonist sensitivity. Similarly, mutating most PI(4,5)P_2_-interacting residues also made it easier for TRPM3 to be inhibited by Gβγ. This is likely to be an allosteric effect, as the Gβγ binding peptide in TRPM3 (12) is located far away from the PI(4,5)P_2_ binding site (Fig. S9). Interestingly, the R1131Q mutant did not display any Gβγ inhibition, pointing to the complex role of this residue in channel regulation.

In contrast to the apparent allosteric interaction between PI(4,5)P_2_ and agonist or Gβγ, disease-associated gain-of-function mutations in TRPM3 that increased both heat and agonist sensitivity (15) did not decrease sensitivity for inhibition by PI(4,5)P_2_ depletion (Figure 9), indicating that the mechanism of increased channel activity is not the consequence of increased sensitivity to PI(4,5)P_2_.

In an earlier work, well before structures became available, three residues in the TRP domain of TRPM8 were proposed to act as PI(4,5)P_2_-interacting residues (36). While mutations in all three of them decreased PI(4,5)P_2_ apparent affinity (36), only one of them was in direct contact with PI(4,5)P_2_ in the TRPM8-PI(4,5)P_2_ structures that were determined later (28). Also before TRP channel structures became available, a short “PH domain–like” segment with several positively charged residues was proposed to act as a PI(4,5)P_2_ interaction site in TRPM4 (53). Even though mutations in this segment behaved in a way compatible with reduced PI(4,5)P_2_ interactions, this segment was far away from the plasma membrane in the subsequently determined TRPM4 structures, which is incompatible with acting as a PI(4,5)P_2_-interacting domain (49). It was also proposed that similar, nonconserved, and short charged amino acid segments are responsible for the effects of PI(4,5)P_2_ on other TRP channels, including TRPM3 and TRPM8, but for channels other than TRPM4, no experimental testing was performed with mutants in those segments (54). The proposed segments are at different locations in different TRPM channels, suggesting that the activation mechanism by PI(4,5)P_2_ is not conserved between different TRPM channels. Our data showing that the PI(4,5)P_2_ binding site in TRPM3 is similar, yet not identical to that in TRPM8 suggests that the PI(4,5)P_2_ binding site in TRPM channels shows substantial level of conservation.

In our earlier work, we used a homology model, based on the structure of TRPV1 combined with mutagenesis, to predict PI(4,5)P_2_-interacting residues in the epithelial Ca^2+^ channel TRPV6 (55). Our homology model–based PI(4,5)P_2_ site was very similar to the experimentally determined PI(4,5)P_2_ binding site in TRPV5, with the same key contact residues (55, 56). TRPV5 and TRPV6 are products of a relatively recent gene duplication, and they share 75% identity, and they are functionally far more similar to each other than to other members of the TRPV subfamily. This gives us confidence that our computationally determined PI(4,5)P_2_ binding site in TRPV6 likely reflects the actual PI(4,5)P_2_ binding site with a reasonable accuracy, even if there is no TRPV6 PI(4,5)P_2_ costructure available currently. Similarly, in the current work, we docked PI(4,5)P_2_ to the apo structure of TRPM8 (30), which showed a high level of overlap with the PI(4,5)P_2_ binding site of TRPM8 determined subsequently by cryo-EM studies (28). This makes us confident that our experimentally tested computational prediction of the PI(4,5)P_2_ binding site in TRPM3 reflects the functionally relevant PI(4,5)P_2_ binding site with reasonable accuracy.

In conclusion, our data provide mechanistic insight into regulation of TRPM3 by its key physiological cofactor, PI(4,5)P_2_. We identify its binding site on the channel, characterize the interaction between PI(4,5)P_2_ and other physiological regulators of TRPM3, and compare its regulation by PI(4,5)P_2_ to that of TRPM8.

## Experimental procedures

### *Xenopus laevis* oocyte preparation and RNA injection

All procedures of preparing *X. laevis* oocytes were approved by the Institutional Animal Care and Use Committee at Rutgers New Jersey Medical School. Frogs were anesthetized in 0.25% ethyl 3-aminobenzoate methanesulfonate solution (pH 7.4) (MS222; Sigma–Aldrich), then bags of ovaries were surgically collected, and rotated with 0.1 to 0.3 mg/ml type 1A collagenase (Sigma–Aldrich) at 16 °C overnight in OR2 buffer containing 82.5 mM NaCl, 2 mM KCl, 1 mM MgCl_2_, and 5 mM Hepes (pH 7.4). Afterward, oocytes were washed with OR2 several times and then kept in OR2 solution supplemented with 1.8 mM CaCl_2_, 100 IU/ml penicillin, and 100 μg/ml streptomycin at 16 °C.

To express exogenous proteins, RNA was microinjected into oocytes using a nanoliter-injector system (Warner Instruments). RNA was *in vitro* transcribed from the linearized pGEMSH vectors, which contained the complementary DNA clones for hTRPM3 (38) rat TRPM8, human M2 muscarinic (hM2) receptor, or Gβγ subunits by using the mMessage mMachine T7 Transcription Kit (Thermo Fisher Scientific). TRPM3 and TRPM8 mutants, which were used in this article, were generated by the QuikChange II XL Site-Directed Mutagenesis Kit from Agilent, and the mutated DNA constructs were confirmed by DNA sequencing. For coexpression of TRPM3 constructs and Gβ1γ2 subunits, 40 ng of TRPM3 was coinjected with 5 ng Gβ1 and 5 ng Gγ2. In the case of coexpressing TRPM3 and hM2 receptors, these two were injected at 1:1 ratio, 40 ng each. Oocytes were used for electrophysiological experiments after 48 to 72 h incubation at 16 °C after RNA injection.

### TEVC experiments

Oocytes were placed in extracellular solution, which contained 97 mM NaCl, 2 mM KCl, 1 mM MgCl_2_, and 5 mM Hepes, pH 7.4. Currents were measured with a protocol consisting a voltage step from the 0 mV holding potential to −100 mV, followed by a ramp to 100 mV once every 0.5 s with a GeneClamp 500B amplifier and analyzed with the pClamp 9.0 software (Molecular Devices). Currents were recorded by thin wall glass pipettes that contained inner filament and were filled with 1% agarose in 3 M KCl. In all TEVC experiments, different concentrations of PregS were applied to activate TRPM3 channels, and various concentrations of menthol were used to trigger responses of TRPM8 channels. The hM2 receptor was activated by 5 μM acetylcholine. For wortmannin experiments specifically, PregS, or menthol-induced currents were measured, then the same oocyte was incubated with 35 μM wortmannin for 2 h, and currents were measured again using the same protocol. In the bar graphs in Figure 3, the individual panels show experiments that were performed on the same day.

### Excised inside–out patch clamp electrophysiology

Oocytes were placed in a recording chamber filled with bath solution, which contained 97 mM KCl, 5 mM EGTA, 10 mM Hepes, pH 7.4. Before starting measurements, the vitelline layer was carefully removed with forceps without damaging the oocyte. Then a giga-ohm seal was formed using a borosilicate glass pipette (World Precision Instruments) with resistance from 0.8 to 1 MΩ. The pipette was filled with a solution containing 97 mM NaCl, 2 mM KCl, 1 mM MgCl_2_, 5 mM Hepes, and 100 μM PregS at pH 7.4. Currents were measured by an Axopatch 200B amplifier and analyzed with the pClamp 9.0 software. Compounds were dissolved in the bath solution and delivered to the inner side of cell membrane by a custom-made gravity-driven perfusion system. Either 25 μM PI(4,5)P_2_, 25 μM PI(4)P, or 10 μM AASt PI(4,5)P_2_ was applied in these experiments to reactivate TRPM3. At the end of every recording, 30 μg/ml Poly-Lys (Poly-K) was applied.

### Maintenance and transfection of human embryonic kidney 293 cells

Human embryonic kidney 293 cells were purchased from American Type Culture Collection (catalog number: CRL-1573). Human embryonic kidney 293 cells were cultured in minimum essential medium supplemented with 10% fetal bovine serum and 100 IU/ml penicillin plus 100 μg/ml streptomycin. Cells were incubated in 5% CO_2_ at 37 °C. Cells were tested to confirm that they were not infected by mycoplasma. Cells were used up to 25 passages and then discarded. Cells were transiently transfected with complementary DNA encoding different TRPM3 constructs (200–400 ng) using the Effectene reagent (Qiagen). mTRPM3α2 and its mutant were cloned into the bicistronic pCAGGS/IRES-GFP vector. The components of rapamycin-inducible pseudojanin phosphatases (40) were cotransfected with mTRPM3α2 at 1:1 ratio.

### Whole-cell patch-clamp experiments

After 24 h of transfection, cells were plated on poly-d-lysine–coated 12 mm cover slips. Experiments were performed 48 to 72 h after transfection. Coverslips were placed in recording chamber filled with extracellular solution (137 mM NaCl, 5 mM KCl, 1 mM MgCl_2_, 10 mM Hepes, and 10 mM glucose, pH 7.4). Since mTRPM3 constructs were in the background of bicistronic pCAGGS/IRES-GFP vector and rapamycin-inducible phosphatases were labeled with red florescent protein, cells that showed both GFP and red florescent protein fluorescence were selected for the whole-cell patch-clamp experiments. Patch pipettes were prepared from borosilicate glass capillaries (Sutter Instruments) using a P-97 pipette puller (Sutter Instrument) with a resistance of 2 to 4 MΩ. Those recording pipettes were filled with intracellular solution containing 140 mM potassium gluconate, 5 mM EGTA, 1 mM MgCl_2_, 10 mM Hepes, and 2 mM Na-ATP, pH 7.4. After formation of gigaohm-resistance seals, the whole cell configuration was established, and currents were recorded by applying a ramp protocol once every 1 s. The holding potential was 0 mV; followed by a −100 mV step for 100 ms; plus a ramp protocol from −100 mV to +100 mV over the period of 500 ms. All recordings were made with an Axopatch 200B amplifier, filtered at 5 kHz, and digitized through a Digidata 1440A interface. Data were collected and analyzed with the pClamp10.6 (Clampex) acquisition software (Molecular Devices) and further analyzed and plotted with Prism 9 (GraphPad by Dotmatics). TRPM3 channels were activated by PregS, and 100 nM of rapamycin was applied to activate phosphatases.

### Statistics

Statistical analysis was performed with Origin 2021 and GraphPad Prism 9. Data were plotted as mean ± SEM and scatter plots or mean ± SD when scatter plots are not provided. Sample sizes were not predetermined by any statistical method; however, they were similar to what is generally used in the field. All recordings were performed in random order. Statistical significance was evaluated with *t* test, or ANOVA with Bonferroni’s post hoc test, or the Kolmogorov–Smirnov nonparametric test, using GraphPad Prism 9, as described in the figure legends. *p* Values are reported in figures or figure legends.

### TRPM8 experimental structure refinement and molecular docking of full-length PI(4,5)P_2_

The cryo-EM structure of full-length apo TRPM8 from *Ficedula albicollis* (PDB ID: 6BPQ) (30), which contains several unresolved amino acid ranges (∼4.1 Å resolution) as well as protein residues with missing atoms, was used as the starting configuration to generate a refined structural model of the TRPM8 channel. The Prime Loop Prediction (57) program and the Protein Preparation Wizard (58) (both distributed by Schrödinger, LLC, 2018) were used to perform the following tasks: (1) loop refinement by serial loop sampling, at the ultraextended accuracy level. In particular, four unresolved amino acid ranges in the transmembrane region were sampled, including 714 to 722, 819 to 822, 889 to 895, and 976 to 990 (sequence numbering as in *F. albicollis*); (2) side-chain prediction of protein residues with missing atoms, performed with no backbone sampling; (3) p*K*a prediction of protein residues at pH 7, followed by analysis and optimization of hydrogen-bond networks; (3) structure refinement *via* restrained minimization of heavy atoms (hydrogens not restrained) using the OPLS (59) force field. The minimization convergence criterion was set to 0.30 Å RMSD for heavy atom displacement. The resulting apo TRPM8 structure was then searched for putative ligand-binding sites using SiteMap (35). Residues facing the topmost suitable site for ligand-binding spot were used to define the docking space for putative PI(4,5)P_2_ binding modes. The program Glide (60) (Schrödinger, LLC, 2018) was used to dock PI(4,5)P_2_ against TRPM8, using a rigid-receptor and flexible-ligand protocol. The ligand was prepared by using the default protocol of LigPrep (Schrödinger, LLC, 2018). Binding modes were ranked using the Glide standard precision scoring function. The best binding mode of PI(4,5)P_2_ against TRPM8 is shown in Figure 2. After our refined TRPM8–PI(4,5)P_2_ complex was generated and used for subsequent modeling of the TRPM3 channel as in a complex with PI(4,5)P_2_, seven additional experimental structures of TRPM8 became available (Table S1). Three of these structures report the TRPM8 channel in complex with PI(4,5)P_2_ as well as Ca^2+^ ions and/or small-molecule ligands (28).

### TRPM3 homology model and molecular docking of a truncated PI(4,5)P_2_ molecule

No experimental structure of the TRPM3 channel is currently available. The cryo-EM structure of the TRPM4 channel (3.1 Å resolution) in the apo state with short coiled coil from *Mus musculus* (PDB ID: 6BCJ) (29) was selected as the template to build a homology model of the hTRPM3 structure using the Swiss-Model Server (61) (https://swissmodel.expasy.org/), based on the human sequence UniProtKB: Q9HCF6. The choice of the template is exemplified in Scheme S1. Essentially, the closest relative to TRPM3 in the TRPM family (cladogram) with an available structural template was selected, that is, TRPM4 (29). The cladogram was generated using Clustal Omega (https://www.ebi.ac.uk/), upon performing a multiple sequence alignment (default settings) (62). The Swiss-Model–generated protein structure of apo TRPM3 was then prepared for subsequent calculations using the Protein Preparation Wizard (58). Potential hot spots for PI(4,5)P_2_ binding to TRPM3 were defined by combining binding-site mapping results obtained using SiteMap (35) with sequence and structure alignments between the refined structural model of TRPM8 in complex with PI(4,5)P_2_ and the TRPM3 model (apo state) generated using Swiss-Model (61). Hence, the TRPM3 protein residues facing the most “druggable” binding spot were selected by homology and used to center the docking grid for subsequent docking of PI(4,5)P_2_. The best binding mode of a truncated version of PI(4,5)P_2_ against TRPM3 is shown in Figure 1. As a matter of fact, because of the extreme flexibility of the lipid tail, the docking algorithm failed in generating binding poses for the full-length PI(4,5)P_2_ lipid. Instead, starting from the PI(4,5)P_2_ headgroup, a series of truncated versions of a growing lipid were docked successfully against the binding site on TRPM3 until a maximum tail length was reached (our truncated lipid is similar to the synthetic diC_8_ PI(4,5)P_2_ molecule, which is experimentally functional in activating TRPM3 (21)). For simplicity, in this work, the PI(4,5)P_2_ lipid with truncated tails, which was modeled in complex with TRPM3, is referred to as PI(4,5)P_2_. Note that in for TRPM8, the PI(4,5)P_2_ molecule was modeled as a full-length lipid. As for the molecular docking, we used the same protocol implemented for TRPM8. Related figures were generated using the Visual Molecular Dynamics (VMD) molecular visualization program (63) (http://www.ks.uiuc.edu/).

### Comparisons of TRPM3 and TRPM8 structural models

A number of structural alignments were performed to compare TRPM3 and TRPM8 structures, including models (TRPM3 and TRPM8) and experimental structures (TRPM8). Superposition of the atomic coordinates was all performed based on sequence alignments (using the algorithm Needelman–Wunsch with BLOSUM-62 matrix). Alignments were generated using the Match Maker tool in UCSF Chimera (64), version 1.15, and analyzed in VMD. A number of structural alignments were performed, described as follows. (1) The model of TRPM8 in complex with (full length) PI(4,5)P_2_ and that of TRPM3 in complex with (truncated) PI(4,5)P_2_ was aligned. (2) The TRPM8/PI(4,5)P_2_ model and the experimentally determined structure of TRPM8 in complex with the menthol analog WS-12 and PI(4,5)P_2_ (PDB ID: 6NR2), and the complex of TRPM8 with icilin (PDB ID: 6NR3), PI(4,5)P_2_, and calcium (28). Pairwise backbone RMSD values were calculated for two separate selections (Table 1), including amino acid ranges facing the lipid binding sites, using the VMD RMSD Trajectory Tool. Before RMSD was calculated, structures were aligned on each selection. The first selection (sel-1 in Table 1) included residues 670 to 685 (on pre-S1), residues 724 to 735 (on pre-S1), and residues 851 to 865 (on linker). The second selection (Sel-2 in Table 1) included residues 670 to 685 (on pre-S1), residues 724 to 735 (on pre-S1), residues 851 to 865 (on linker), and residues 997 to 1009 (on TRP domain). (3) The following structures were aligned to the TRPM3–PI(4,5)P_2_ model: the experimental structure of TRPM4 (29) and TRPM3 models from AlphaFold (DeepMind, EMBL-EBI) (31, 32). At the time of writing, four AlphaFold models were available of TRPM3, each from a different organism (UniProt sequence ID: Q9HCF6 [human; Fig. S1], J9S314 [*M. musculus*], F1QYX6 [*Danio rerio*], and F1LN45 [*Rattus norvegicus*]). All four structures were superimposed (not shown), revealing striking structural similarities. All figures related to (1) to (3) were generated using VMD.

### ΔΔG calculations

Changes in the binding affinity (or Gibbs free energy of binding, ΔΔ*G* in kcal/mol) of PI(4,5)P_2_ to TRPM3 were calculated upon mutating key binding residues in the putative PI(4,5)P_2_ binding site. These residues were also mutated experimentally. To do so, a physics-based scoring was employed (65), previously used with systems similar to the one included in this study (25, 66). Essentially, residue mutations and ΔΔ*G* calculations were performed on the TRPM3 model bound to truncated PI(4,5)P_2_ molecules as generated from molecular docking, that is, the native structural complexes or WT. A total of three binding modes were used as native configurations, including the TRPM3 model bound to the truncated PI(4,5)P_2_ presented in this study, and two additional poses with different protonation states of the phospholipid headgroup and even shorter tails. Then, the “Residue-Scanning and Mutation” tool from BioLuminate (65) (Schrödinger, LLC, 2018) was used to perform calculations upon mutating the native system, as described in Table S2. For each of the three WT proteins, six additional mutants were generated, reaching a total of 21 systems. For each system, and for both the WT and the mutant, an MM/GBSA refinement of the bound and unbound states was performed using Prime (Schrodinger, LLC, 2018), *via* the VSGB 2.0 implicit continuum solvation model (43). In VSGB 2.0, the solvation free energy is approximated with an optimized model based on the surface generalized Born method (44) and the variable dielectric treatment of polarization (45) for protein residues. The latter incorporates the polarization effects by changing the value of the internal dielectric constant (from 1.0 to 4.0) (43). No implicit membrane model (*i.e.*, a low-dielectric slab) was used, and therefore, the results should be regarded as an approximation to the electrostatic energy.

The structural complexes were refined by side-chain prediction with backbone sampling/minimization of the mutated residue, before a minimization in the region around the mutation site was performed to relax and optimize the side-chain interactions with the lipid. Systems were prepared for the calculations using the Protein Preparation Wizard (58).

A thermodynamic cycle was then used to calculate the change in the binding affinity, ΔΔG(bind), of a protein upon single amino acid mutation, as represented below:

**Figure:**
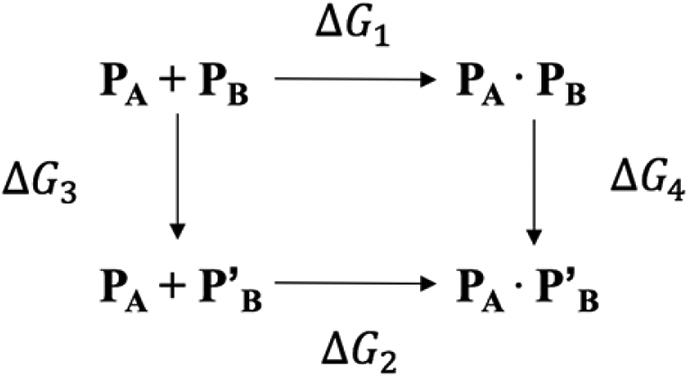

where P_A_ and P_B_ are, respectively, the ligand and the receptor binding partners in the parent, and P’_B_ is the mutated binding partner. While P_A_ + P_B_ and P_A_ + P’_B_ are the separated binding partners, P_A_ · P_B_ and P_A_ · P’_B_ are the bound partners.

The change in the binding affinity (the net ΔΔG free energy difference) was calculated by addressing the free energy changes in *vertical lines*, easier to simulate than the experimental observables (*horizontal lines*).

(1) $$\Delta \Delta G_{bind}=\Delta G_{2}-\Delta G_{1}=\Delta G_{4}-\Delta G_{3}$$

A positive value of $\Delta \Delta G_{bind}$ indicates that the WT binds better than the mutant. Affinity changes were plotted using Microsoft Excel (https://www.microsoft.com/). Related figures were generated using VMD.

## Data availability

All data are contained in the article and supporting information. The structural model of TRPM3 in complex with a PI(4,5)P_2_ phospholipid with short tails is also available as a supporting information file. The authors request that any published work derived from the use of such data include a reference to this publication.

## Supporting information

This article contains supporting information.

## Conflict of interest

The authors declare that they have no conflicts of interest with the contents of this article.

## Supporting information

Supporting information

Supplemental Scheme 1 and Fig. S1–S9 and Table S1–S2
