# Supplementary material for: Computational and functional studies of the PI(4,5)P2 binding site of the TRPM3 ion channel reveal interactions with other regulators
Source: J Biol Chem. 2022 Sep 28;298(11):102547. doi: 10.1016/j.jbc.2022.102547 (PMC9647539; doi:10.1016/j.jbc.2022.102547)
Supplement: Supplemental Scheme 1 and Fig. S1–S9 and Table S1–S2 [file mmc2.docx]

Supplemental File to:

Computational and functional characterization of the PI(4,5)P_2_ binding site of the TRPM3 ion channel

Siyuan Zhao (1), Vincenzo Carnevale (2,3), Matthew Gabrielle (1), Eleonora Gianti (2,4)*, Tibor Rohacs (1)*

(1) Department of Pharmacology, Physiology and Neuroscience, Rutgers, New Jersey Medical School, Newark, NJ 07103

(2) Institute for Computational Molecular Science, (3) Department of Biology, (4) Department of Chemistry, Temple University, Philadelphia, PA 19122

*Correspondence: [tibor.rohacs@rutgers.edu](mailto:tibor.rohacs@rutgers.edu), or [egianti@temple.edu](mailto:egianti@temple.edu)

[https://doi.org/10.1101/2022.06.05.494899](https://nam02.safelinks.protection.outlook.com/?url=https%3A%2F%2Fdoi.org%2F10.1101%2F2022.06.05.494899&data=05%7C01%7Crohacsti%40njms.rutgers.edu%7C344b429898944bd345fb08da766a069e%7Cb92d2b234d35447093ff69aca6632ffe%7C1%7C0%7C637952495086584297%7CUnknown%7CTWFpbGZsb3d8eyJWIjoiMC4wLjAwMDAiLCJQIjoiV2luMzIiLCJBTiI6Ik1haWwiLCJXVCI6Mn0%3D%7C3000%7C%7C%7C&sdata=lKyVHIqpAI6ioSQaNf17tXYuJGlpUmUoSH201qitNqg%3D&reserved=0)

Structural model of TRPM3 in complex with a phospholipid (PI(4,5)P2 with short tails).

The authors request that any published work derived from the use of such data include a reference to this publication.
