# Supplementary material for: Computational and functional studies of the PI(4,5)P2 binding site of the TRPM3 ion channel reveal interactions with other regulators
Source: J Biol Chem. 2022 Sep 28;298(11):102547. doi: 10.1016/j.jbc.2022.102547 (PMC9647539; doi:10.1016/j.jbc.2022.102547)
Supplement: Supplemental File [file mmc3.pdf]

Computational and functional studies of the PI(4,5)P_2 binding site of the TRPM3 ion channel reveal interactions with other regulators

Siyuan Zhao[1], Vincenzo Carnevale[2,3], Matthew Gabrielle[1], Eleonora Gianti[2,4*], Tibor Rohacs[1*]

SUPPORTING INFORMATION

List of material:

Scheme 1
Figures S1-S9
Tables S1-S2

Structural model of TRPM3 with PI(4,5)P_2 (pdb) is in a separate file

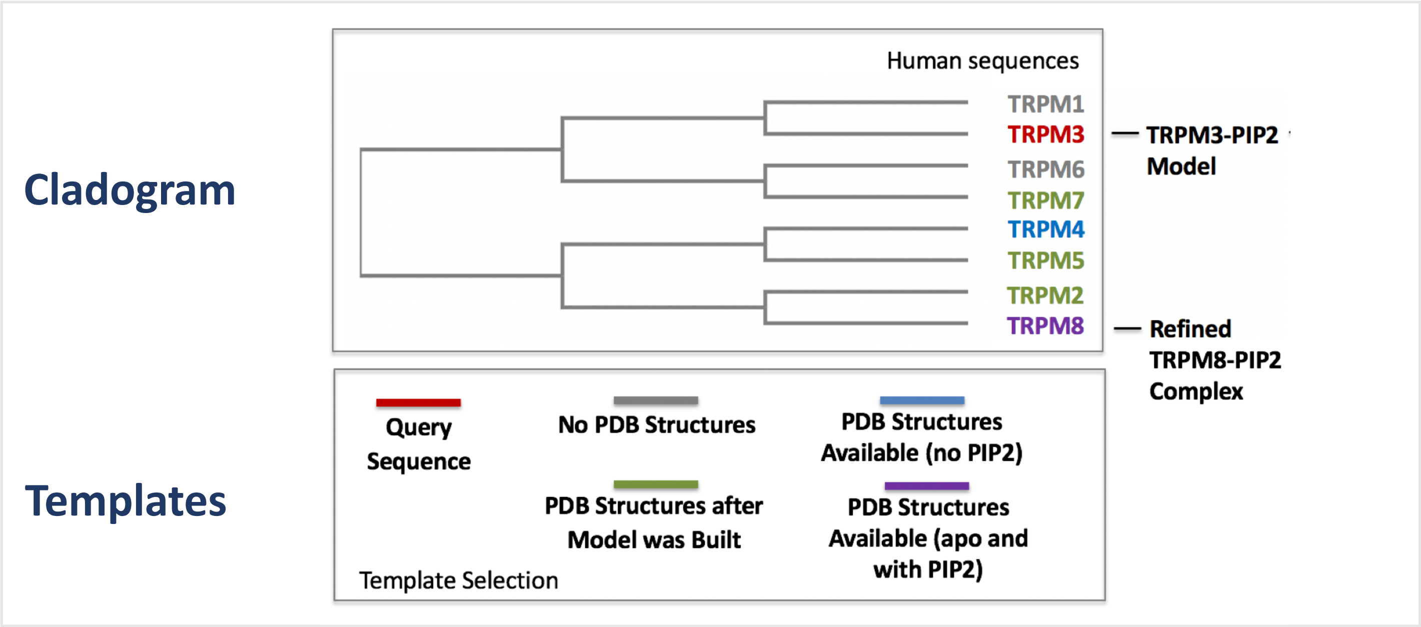

Scheme S1. Cladogram and inventory of experimental structures available as templates for building the model of TRPM3 in complex with PI(4,5)P_2. When the model was built, the structure of TRPM4 in a ligand free state was used as the template (PDB-ID 6BCJ) (29). This structure was selected as the closest homolog to TRPM3 in the TRPM family with an experimental structure available at the time.

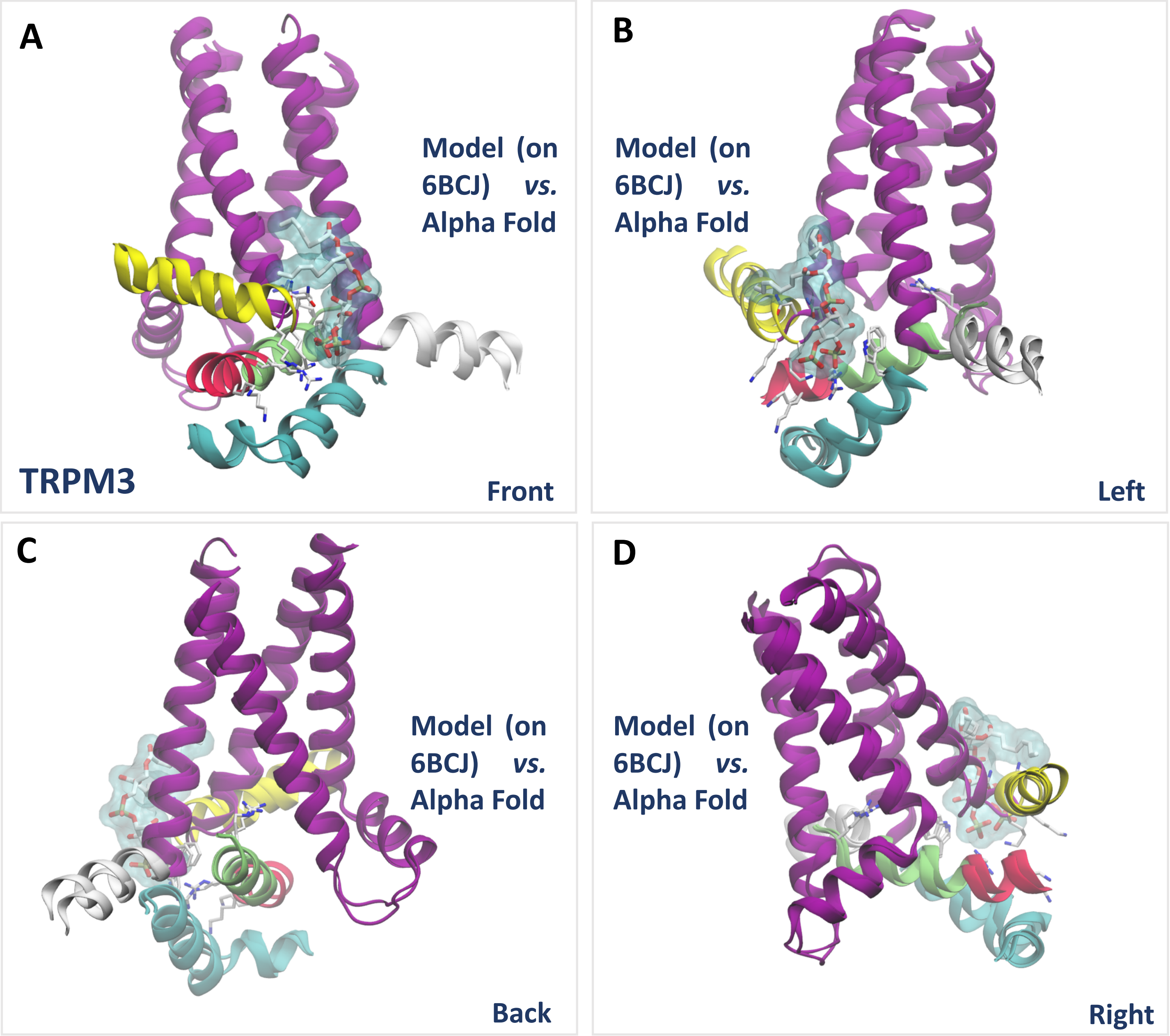

Figure S1. Structural comparisons of the TRPM3 model in complex with PI(4,5)P_2 with a ligand-free model from AlphaFold. (**A to D**) Close-up views of structural superposition of the PI(4,5)P_2 binding site in two different TRPM3 structures, namely the TRPM3 model (built on TRPM4, 6BCJ) (29) in complex with PI(4,5)P_2, which was built in this work, and the ligand-free protomer of human TRPM3 from AlphaFold (Q9HCF6) (31). All representations are reproduced as in **Figure 1**.

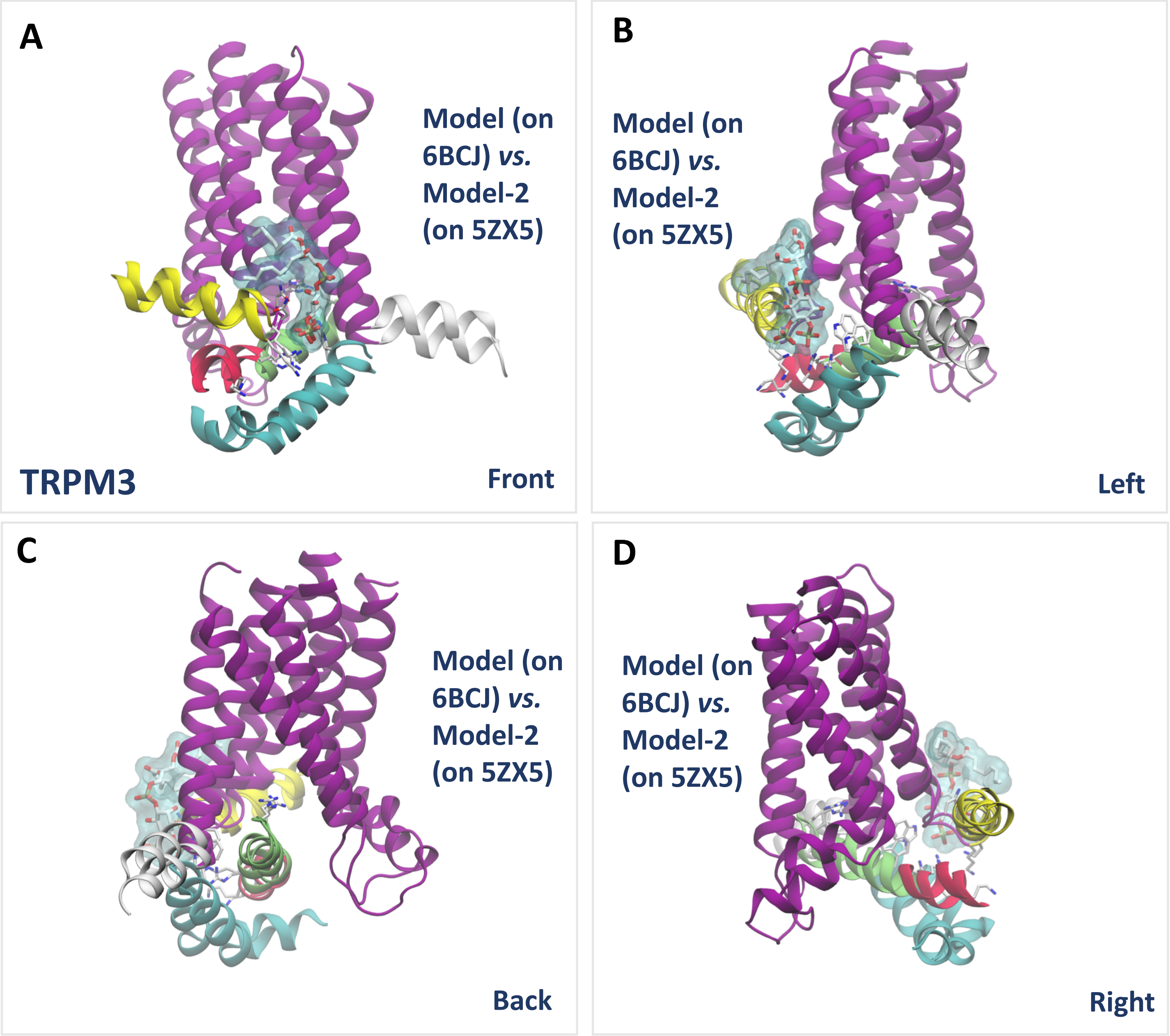

Figure S2. Structural comparison of TRPM3 models. (**A to D**) Close-up views of structural superposition of the PI(4,5)P_2 binding site in two different TRPM3 models (both built in this work). The first is the main TRPM3 model in complex with PI(4,5)P_2, built using TRPM4 (6BCJ) (29) as the template. The second is a model of ligand-free TRPM3 obtained using the cryo-EM structure of TRPM7 in EDTA (5ZX5) (33) as the template. All representations are reproduced as in **Figure 1**.

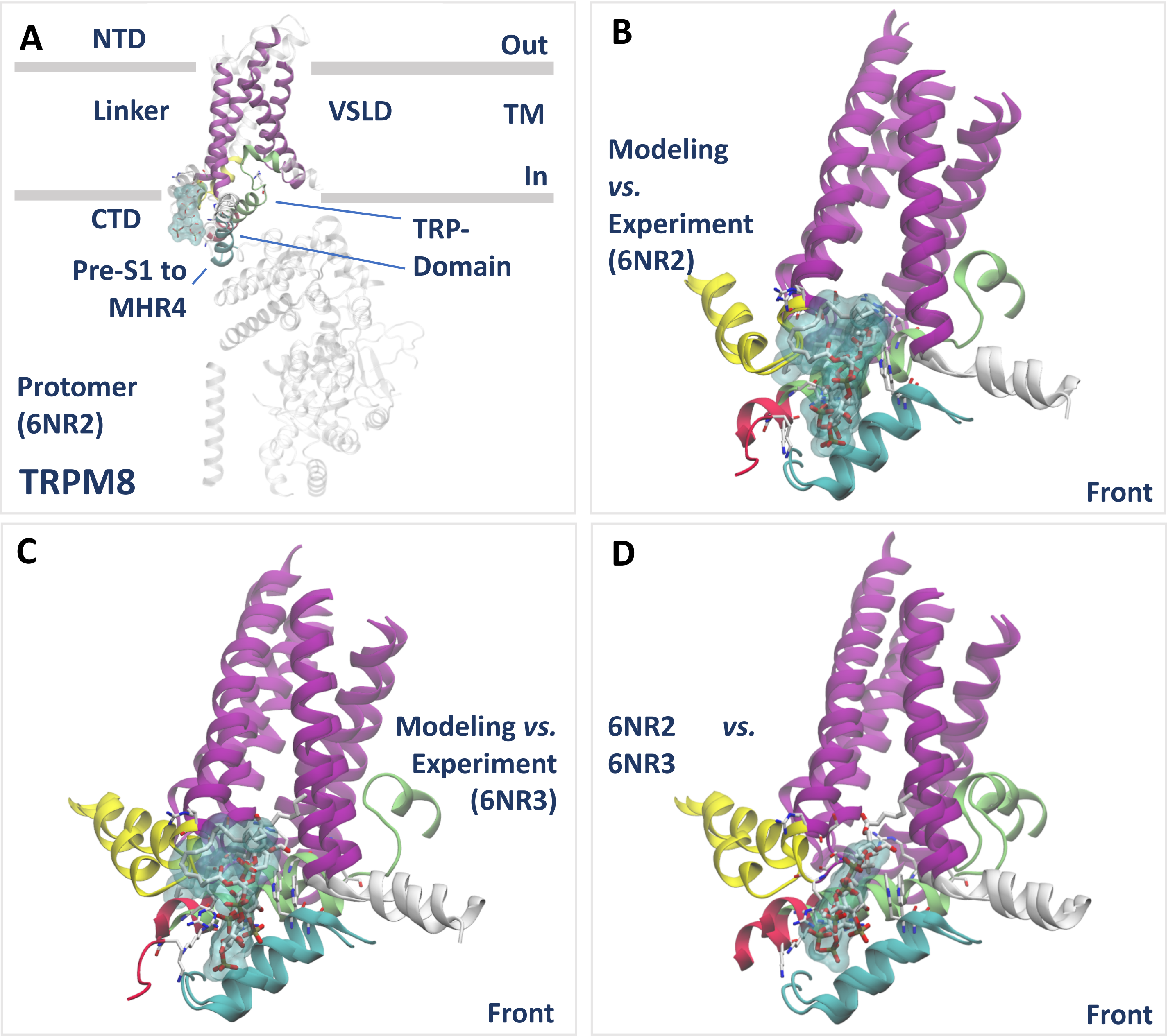

Figure S3. Structural comparisons of the model of TRPM8 in complex with PI(4,5)P_2 with experimental structures of TRPM8. (**A**) Protomer view from the transmembrane (TM) plane of the cryo-EM structure of TRPM8 in complex with the menthol analog WS-12 and PI(4,5)P_2 (6NR2) (28). The structure compares with the refined model of TRPM8 in complex with PI(4,5)P_2 (**Figure 2**). (**B to D**) Close-up views of structural superposition of the PI(4,5)P_2 binding site in different TRPM8 structures. In (B), the refined model of TRPM8 in complex with PI(4,5)P_2 is superposed to the cryo-EM structure shown in (A). In (C), the model is superposed to the cryo-EM protomer of TRPM8 in complex with icilin, PI(4,5)P_2 and calcium (6NR3) (28). In D, protomers from the two cryo-EM structures of TRPM8 in complex with PI(4,5)P_2 are superposed. NTD, N-terminal domain; CTD, C-terminal domain. All representations are reproduced as in **Figure 1**.

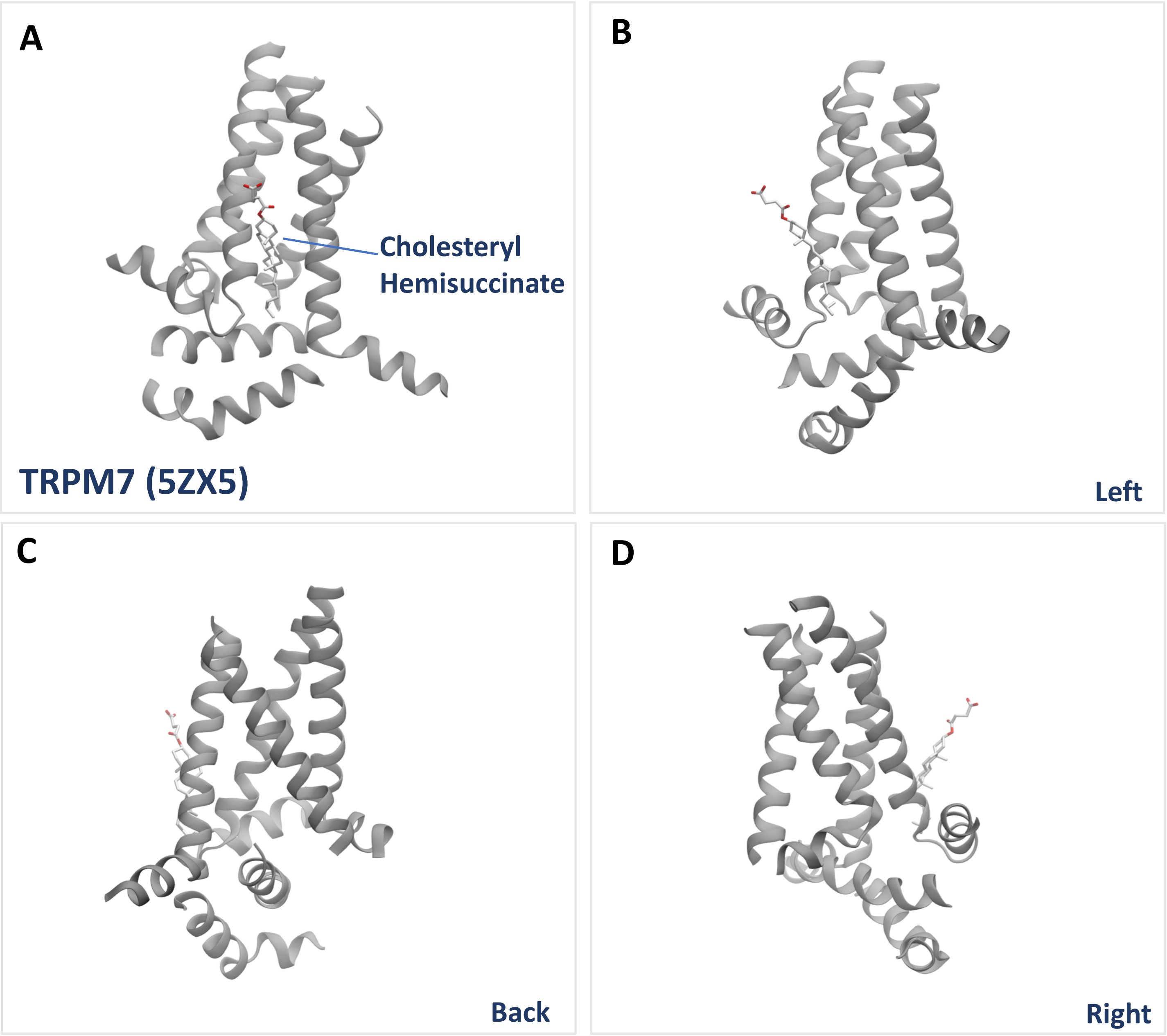

Figure S4. Lipid binding site in the experimental structure of TRPM7. (**A to D**) Close-up views of a putative lipid binding site in the cryo-EM structure of TRPM7 (5ZX5) (33). The site superposes well with the PI(4,5)P_2 binding site identified in our TRPM3 model (**Figure 1**), and in our TRPM8 model (**Figure 2**) and in the cryo-EM structure of TRPM8 bound to PI(4,5)P_2 (**Figure S3**). In the cryo-EM structure of TRPM7, a molecule of the detergent cholesteryl-hemisuccinate (CHS) is bound to TRPM7 at the putative lipid binding site. Protein atoms are shown in new cartoon representation, colored in grey. CHS atoms are shown in licorice representation, with C, N, O atoms colored in white, blue and red, respectively.

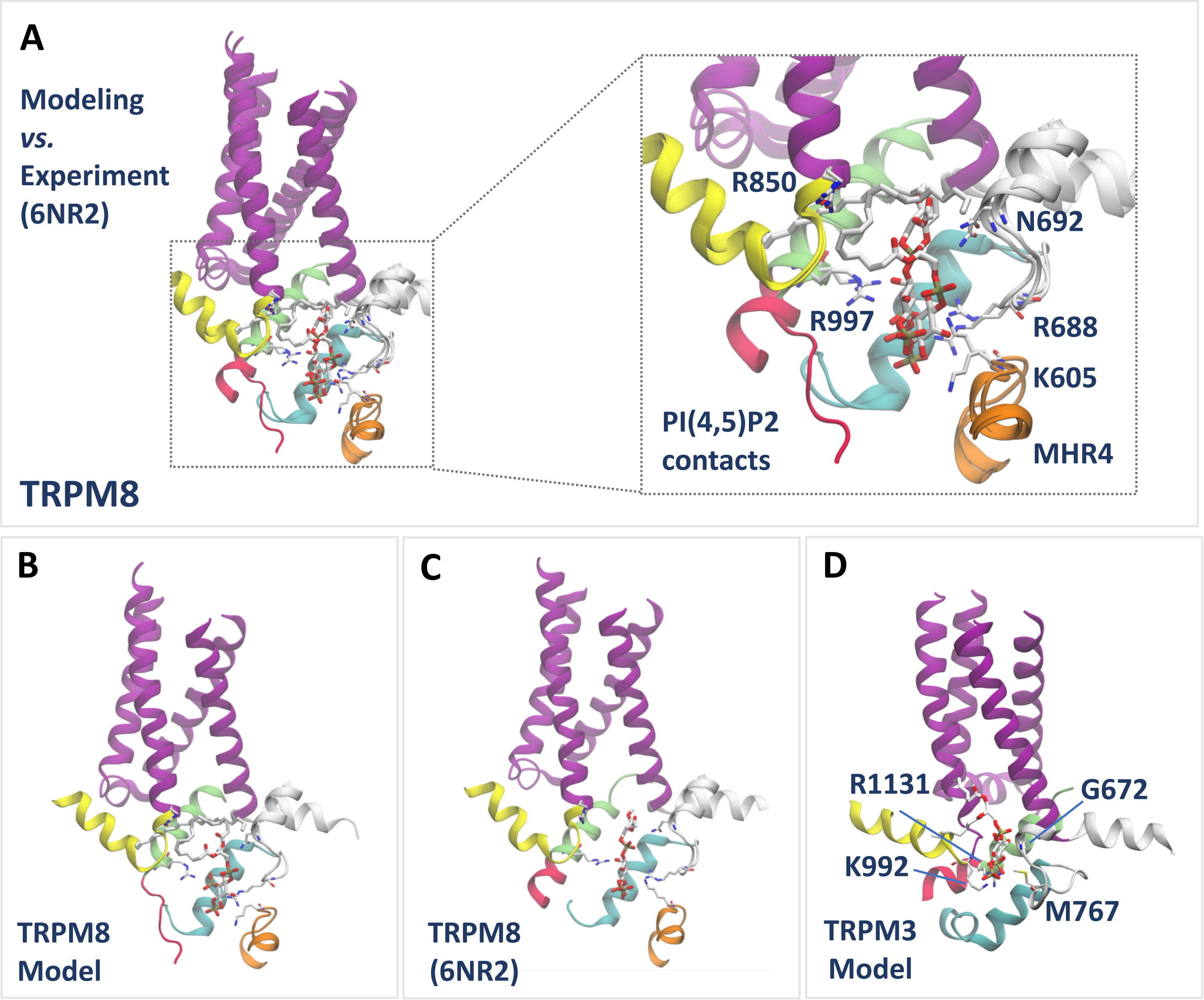

Figure S5. Structural comparisons of the PI(4,5)P$_2$ binding site in TRPM3 and TRPM8 structures. (A) Close-up view with relative zoom-in (inset) of the PI(4,5)P$_2$ binding site in the refined model of TRPM8 in complex with PI(4,5)P$_2$ (**Figure 2**) superposed to the cryo-EM structure of TRPM8 in complex with the menthol analog WS-12 and PI(4,5)P$_2$ (6NR2) (28). In the experimental structure, both WS-12 and PI(4,5)P$_2$ are not shown. The experimentally determined PI(4,5)P$_2$ contact residues (28) are shown in the inset. (**B to D**) Close-up views of (**B**) the refined model of TRPM8 in complex with PI(4,5)P$_2$; (**C**) the structure of TRPM8 in complex with the menthol analog WS-12 and PI(4,5)P$_2$ (6NR2); and (**D**) the TRPM3 model (**Figure 1**) in complex with PI(4,5)P$_2$. The TRPM3 residues equivalent to the experimentally determined PI(4,5)P$_2$ contacts (28) are shown. Comparisons of the PI(4,5)P$_2$ site between TRPM8 and TRPM3 indicate striking structural similarities, with the exception of the MHR4 region (in orange new-cartoon representation), which is not conserved in TRPM3. All representations are reproduced as in **Figure 1**.

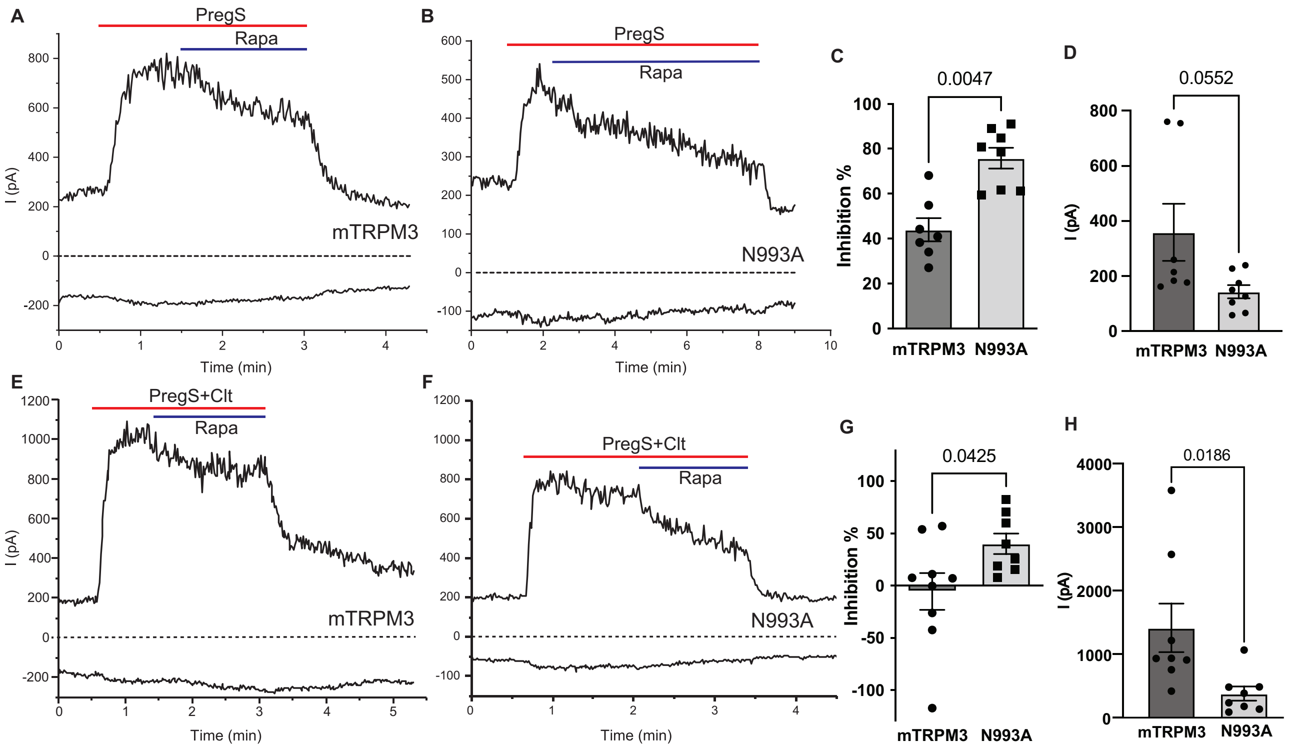

Figure S6. Mutating putative $PI(4,5)P_2$ interacting residues increases sensitivity of TRPM3 to inhibition by $PI(4,5)P_2$ depletion in HEK cells

HEK293 cells were transiently transfected with mouse TRPM3α2 (mTRPM3α2) or its mutant N993A, and the rapamycin inducible pseudojanin phosphatase constructs. Whole cell patch clamp electrophysiology was performed by using ramp protocols from -100 mv to 100 mv as described in the Experimental Procedures section. (A-B) Representative traces of mTRPM3α2 (A) and N993A (B). Applications of 25 μM PregS and co application of 100 nM rapamycin are indicated by red and blue lines respectively. Top traces show currents at +100 mV; dash lines indicate zero current; bottom traces show currents at -100 mV. (C) Data summary of the percentage of inhibition caused by $PI(4,5)P_2$ depletion (rapamycin). Decreased Current amplitudes after rapamycin were normalized to PregS induced current amplitudes. (D) Summary of amplitudes of PregS-induced currents at 100 mV. (E-F) Representative traces of mTRPM3α2 (E) and N993A (F). Applications of 25 μM PregS and 10 μM Clotrimazole (Clt) are indicated by red lines. Applications of 100 nM rapamycin are indicated by blue lines. (G) Data summary of the percentage of inhibition caused by $PI(4,5)P_2$ depletion. Decreased Current amplitudes after rapamycin were normalized to co application of PregS and clotrimazole (Clt) induced current amplitudes. (H) Summary of amplitudes of currents induced by PregS+Clt at 100 mV. Statistical significance was calculated by T test. Bar graphs show mean ± SEM and scatter plots, representing individual measurements from three independent transfections.

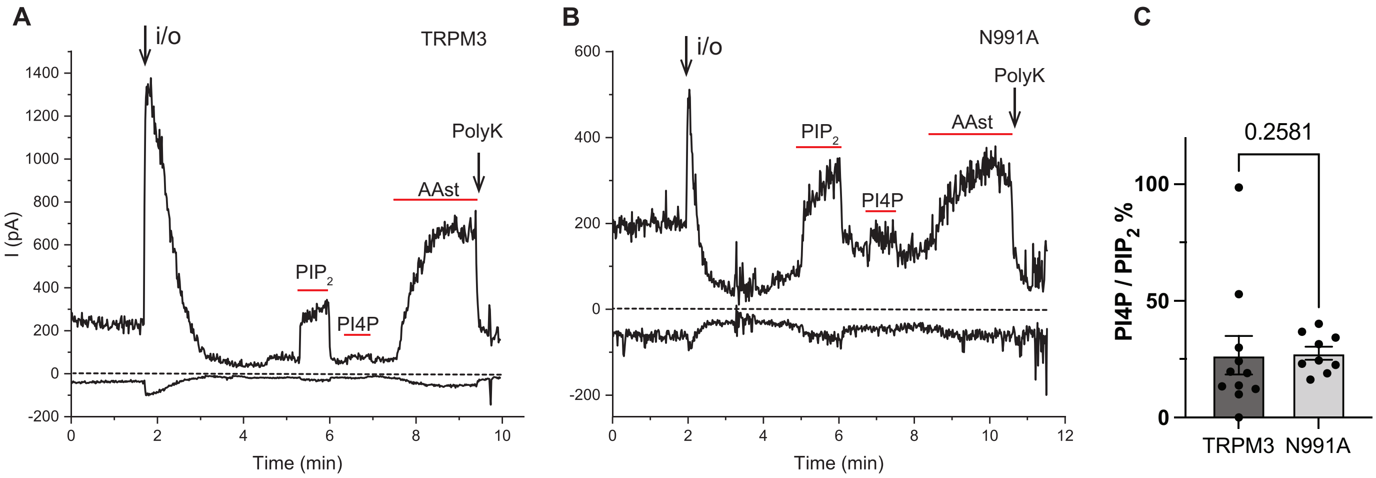

Figure S7. Mutating putative PI(4,5)P_2 interacting residues does not change the relative effect of PI(4)P compared to PI(4,5)P_2

hTRPM3 or its mutant N991A was expressed in oocytes and excised inside-out patch clamp electrophysiology was performed using ramp protocol from -100 mV to 100 mV as described in the Experimental Procedures section. (A-B) Representative traces of hTRPM3 (A) and N991A (B). Top traces show currents at +100 mV; dash lines indicate zero current; bottom traces show currents at -100 mV. The formation of inside out configuration (i/o) is indicated by the arrow. Applications of 25 μM diC_8 PI(4)P, 25 μM diC_8 PI(4,5)P_2 and 10 μM AAst PI(4,5)P_2 are indicated by red lines, 30 μg/ml Poly-Lys (Poly K) was applied at the end and indicated by the second arrow. The patch pipettes contained 100 μM PregS to activate TRPM3 channels. (C) Data summary of the relative effect of PI(4)P compared to PI(4,5)P_2. Current amplitudes induced by PI(4)P was normalized to current amplitudes induced by PI(4,5)P_2. Kolmogorov-Smirnov non-parametric test was used to analyze data and P value was reported on the bar graph.

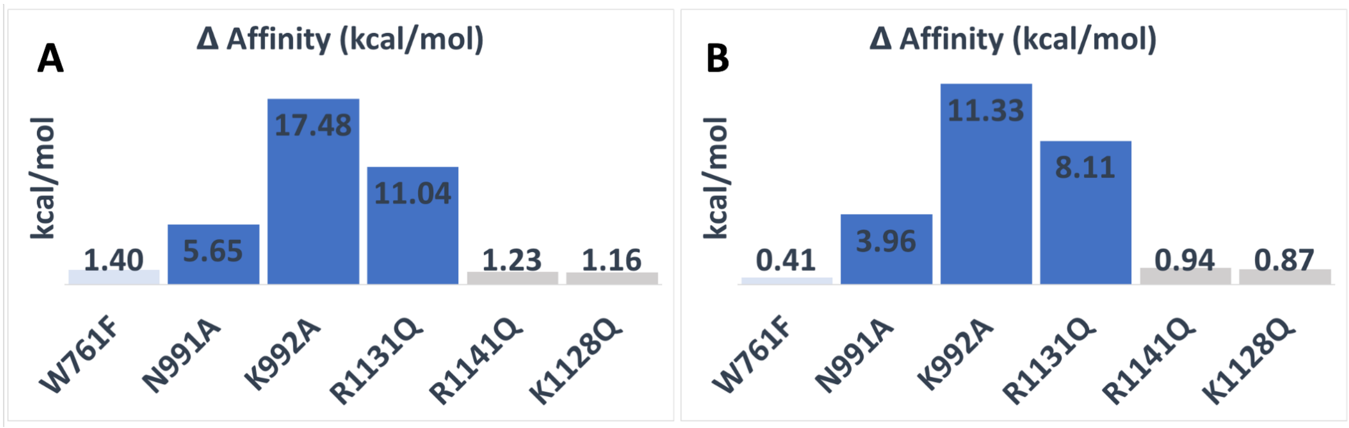

Figure S8. Change in binding affinity (ΔΔG; kcal/mol) upon mutating protein residues *in silico* in the PI(4,5)P_2 binding site of TRPM3, as listed in **Table S2**. In (**A**), PI(4,5)P_2 tail-2 single protonation (tail-2 prot-2); in (**B**), double protonation (Tail-2; prot-3). In blue, mutants that bind significantly worse than the native protein, indicating loss of interaction with PI(4,5)P_2 upon mutations. In light blue, change in the affinity upon mutating W761 to F.
Both the length of the lipid tails and the protonation states of head groups impact the binding mode of the native structure, resulting in a less marked change in the ΔΔG of binding compared to the phospholipid included in the model (**Fig. 4A**). In grey, mutants with no significant effect on binding.

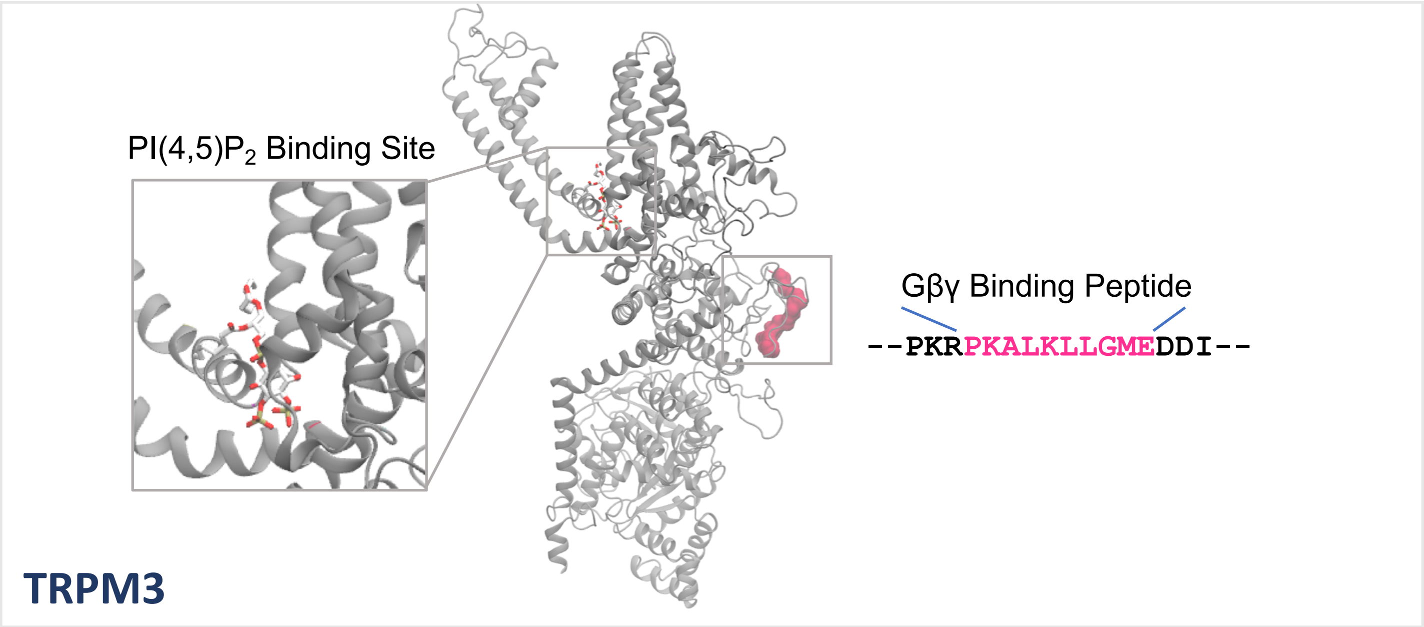

Figure S9. Location of the Gβγ binding peptide on TRPM3. (**Central**) View of a protomer of the TRPM3 model in complex with PI(4,5)P_2, highlighting the location of the Gβγ-binding peptide (bright red) (12). (**Left inset**) A zoom-in view of the PI(4,5)P_2 binding site is shown. (**Right**) The Gβγ-binding peptide sequence (in TRPM3) is shown (bright red). Protein atoms are shown in new cartoon representation, colored in grey. The Gβγ-binding region is shown in surface representation (bright red). PI(4,5)P_2 atoms are shown in licorice representation, with C, N, O atoms colored in white, blue and red, respectively.

PDB-ID	Channel	Release	State
6BPQ	TRPM8	Before TRPM3 model	Ligand free
6NR2	TRPM8	After TRPM3 model	Menthol analog WS-12 and PI(4,5)P2
6NR3	TRPM8	After TRPM3 model	Icilin, PI(4,5)P2, and Ca2+ (high occupancy)
6NR4	TRPM8	After TRPM3 model	Icilin, PI(4,5)P2, and Ca2+ (low occupancy)
6O6A	TRPM8	After TRPM3 model	Ligand free
6O6R	TRPM8	After TRPM3 model	AMTB-bound state
6O72	TRPM8	After TRPM3 model	TC-I 2014-bound state
6O77	TRPM8	After TRPM3 model	Calcium-bound state

Table S1. Experimental structures of TRPM8 available (https://www.rcsb.org). The template selected for generating the refined model of TRPM8 in complex with PI(4,5)P_2 is 6BPQ. This is the only TRPM8 structure released before the TRPM3 model was built (the latter was built on TRPM4 6BCJ as the template).

TRPM3 Mutant (Exp. Isoform)	TRPM3 Mutant (Comp. Isoform)	Δ Affinity (kcal/mol)	PI(4,5)P2 Lipid	Refined TRPM8 Model (on 6BPQ)
W761F	W786F	6.00	Tail-1; prot1	W682
N991A	N1016A	7.90	Tail-1; prot1	S849
K992A	K1017A	16.4	Tail-1; prot1	R850
R1131Q	R1156Q	8.60	Tail-1; prot1	R997
R1141Q	R1166Q	1.10	Tail-1; prot1	R1007
K1128Q	K1153Q	1.20	Tail-1; prot1	K994
W761F	W786F	1.40	Tail-2; prot-2	W682
N991A	N1016A	5.65	Tail-2; prot-2	S849
K992A	K1017A	17.48	Tail-2; prot-2	R850
R1131Q	R1156Q	11.04	Tail-2; prot-2	R997
R1141Q	R1166Q	1.30	Tail-2; prot-2	R1007
K1128Q	K1153Q	1.16	Tail-2; prot-2	K994
W761F	W786F	0.41	Tail-2; prot-3	W682
N991A	N1016A	3.96	Tail-2; prot-3	S849
K992A	K1017A	11.33	Tail-2; prot-3	R850
R1131Q	R1156Q	8.11	Tail-2; prot-3	R997
R1141Q	R1166Q	0.94	Tail-2; prot-3	R1007
K1128Q	K1153Q	0.87	Tail-2; prot-3	K994

Table S2. Change in the binding affinity (Δ affinity or ΔΔG) for PI(4,5)P_2 to the human TRPM3 model (Q9HCF6) predicted upon mutating key residues responsible for binding. Sequence notation for both the experimental and the computational isoforms are reported, as well as the corresponding residues mapped on the TRPM8 binding site (*Ficedula albicollis)*. Tail-1 and -2 indicate phospholipids with tails of different lengths (with tail-2 > tail-1). Different protonation states were modeled (prot-1 to -3). Prot-1 and prot-2 are single protonation states; prot-3 is double protonation.
